# Serum lactate level and mortality in metformin-associated lactic acidosis requiring renal replacement therapy: a systematic review of case reports and case series

**DOI:** 10.1186/s12882-017-0640-4

**Published:** 2017-07-10

**Authors:** Hung-Chieh Yeh, I-Wen Ting, Ching-Wei Tsai, Jenn-Yu Wu, Chin-Chi Kuo

**Affiliations:** 1 Kidney Institute and Division of Nephrology, Department of Internal Medicine, China Medical University Hospital and College of Medicine, China Medical University, 2, Yude Rd., North Dist, Taichung City, 404 Taiwan; 2 Big Data Center, China Medical University Hospital and College of Medicine, China Medical University, Taichung, Taiwan; 3 Department of Internal Medicine, National Taiwan University Hospital Yun-Lin Branch, Yun-Lin, Taiwan

**Keywords:** Metformin, Acute kidney injury, Metabolic acidosis, Renal replacement therapy, Lactate

## Abstract

**Background:**

The current practice concerning timing, mode, and dose of renal replacement therapy (RRT) in patients with metformin-associated lactic acidosis (MALA) with renal failure remains unknown. To investigate whether serum lactate level and prescription pattern of RRT are associated with mortality in patients with MALA requiring RRT.

**Methods:**

We searched PubMed/Medline and EMBASE from inception to Sep 2014 and applied predetermined exclusion criteria. Case-level data including case’s demographics and clinical information related to MALA were abstracted. Multiple logistic regression modeling was used to examine the predictors of mortality.

**Results:**

A total of 253 unique cases were identified with cumulative mortality of 17.2%. Eighty-seven percent of patients had acute kidney injury. Serum lactate level was significantly higher in non-survivors (median 22.5 mmol/L) than in survivors (17.0 mmol/L, *p*-value <0.01) and so did the median blood metformin concentrations (58.5 vs. 43.9 mg/L, *p*-value = 0.05). The survival advantage was not significantly different between the modalities of RRT. The adjusted odds ratio of mortality for every one mmol/L increase in serum lactate level was 1.09 (95% CI 1.02–1.17, *p*-value = 0.01). The dose-response curve indicated a lactate threshold greater than 20 mmol/L was significantly associated with mortality.

**Conclusions:**

Our study suggests that predialysis level of serum lactate level is an important marker of mortality in MALA patients requiring RRT with a linear dose-response relationship. To better evaluate the optimal prescription of RRT in MALA, we recommend fostering an international consortium to support prospective research and large-scale standardized case collection.

**Electronic supplementary material:**

The online version of this article (doi:10.1186/s12882-017-0640-4) contains supplementary material, which is available to authorized users.

## Background

Metformin is recommended as the first-line pharmacological agent for type 2 diabetes by international guidelines for its weight-neutrality and strong evidence-based cost-effectiveness in preventing diabetic vasculopathy [1, 2]. The spectrum of clinical application of metformin has continued to widen and presently include metabolic syndrome and polycystic ovarian syndrome [3, 4]. The most feared adverse effect of metformin use is lactic acidosis due to the historically reported mortality of approximately 50% during the period of 1960–2000 and about 25% since 2000 [5]. Although metformin-associated lactic acidosis (MALA) is rare, with a stable estimated incidence of 1 to 10 per 100,000 [6–8], the stigma concern greatly limit the use of metformin in elderly populations where heart failure and renal impairment are common conditions [9].

The rarity and fatality of MALA make it difficult to obtain conclusive evidence regarding predisposing and prognostic factors as most studies performed to date have been retrospective single-center or registry-based ecological studies with small sample sizes and without blood metformin levels. Consequently, whether to discontinue metformin in patients with cardiac or renal dysfunction remains undetermined and a growing body of evidence demands a critical re-evaluation of contraindications for metformin [7]. More importantly, standard management of MALA is uncertain albeit renal replacement therapy (RRT) has been considered crucial in treating severe form of MALA for its ability to provide renal support, eliminate metformin, and optimize acid-base status [7]. The role of timing, dose, and mode of RRT in MALA is also unknown. To understand the current practice and trend of RRT in MALA, we systematically reviewed all case reports of MALA requiring RRT in the literature worldwide. As the selection of RRT mode is highly institutionalized and inherent spectrum bias in a single-center study, a systematic review of all case reports would be more informative to describe the RRT prescription pattern in MALA. We also evaluated the epidemiological and clinical prognostic factors that associate with mortality of MALA complicated by at least renal failure.

## Methods

### Search strategy and study selection

The systematic search and review processes were conducted in accordance with the Preferred Reporting Items for Systematic Reviews and Meta-Analyses (PRISMA) Statement criteria [10]. We searched PubMed/Medline and EMBASE for case reports of metformin-associated lactic acidosis (MALA) requiring renal replacement therapy by combining three key research concepts derived from relevant MeSH and text-word terms such as “renal replacement therapy”, “dialysis”, “metformin”, and “lactic acidosis”. Additional file 1: Table S1 showed the full search strategy. The search period was January 1966 through Sep 15, 2014. There were no language restrictions. We also manually reviewed the reference lists from relevant review articles and the investigators’ files.

Our exclusion criteria were: 1) reviews, case series, editorials, and research letters that did not provide individual biochemical data; 2) when metformin was not the only offending factor causing lactic acidosis; 3) cases that did not received renal replacement therapy in the disease course; 4) phenformin- or buformin-related lactic acidosis cases; 5) when survival status of an individual patient could not be ascertained. Duplicate publication between conference abstracts and full papers was identified by authors’ group and the demographics of the cases (Additional file 1: Figure S1).

### Data abstraction

Two authors, C.C. Kuo and H.C. Yeh, independently abstracted data from the articles that met the selection criteria. We developed a data extraction form to record the characteristics of the study (authors, journal, years of publication, and country); the characteristics of participants (basic demographics, underlying medical conditions, and outcome data on mortality or end stage renal disease); initial presentations (symptoms and signs); information on biochemical indicators of MALA (peak serum creatinine level, peak blood lactate level, and blood gas measurements); mode and dose of renal replacement therapy (RRT); the causes of death (solely related to MALA or not). In the event of disagreement, other nephrologists, C.W. Tsai and I.W. Ting, were consulted and a consensus was reached.

### Definition of key variables

All information in this review including baseline demographics was directly extracted from the primary literature at individual level. The diagnostic criteria for MALA described in this review refers to an arterial pH less than 7.35 and a serum lactate level above 5 mmol/L in association with metformin exposure, or based on the author’s clinical diagnosis where papers not providing measurements of either pH or lactate level [11]. The baseline renal function was defined as the lowest serum creatinine concentration that could be traced back prior to the index event. Peak serum creatinine and lactate levels were defined as the highest serum creatinine and lactate concentrations, respectively, after the index event and before RRT initiation (Additional file 1: Figure S2). Other clinical parameters related to MALA including pH, serum bicarbonate, and glucose were also abstracted using the reported values closest in time prior to the peak lactate level or the same time. Values greater than the highest bound and less than the lowest bound of the individual’s laboratory reportable range were assigned the highest laboratory reportable value and a half of the detection limit, respectively. Cases with post-MALA ESRD were defined as being long-term RRT dependent after the index event. Cases with acute kidney injury (AKI) was defined either by the authors of the source paper or by an absolute decline from baseline renal function. Inappropriateness of metformin dosing was defined by (1) the contraindicated use of metformin if serum creatinine higher than 1.4 and 1.5 mg/dL in women and men, respectively, based on the US-FDA black-box warning and (2) the prescribed dose higher than the maximal dosage recommended in a recently proposed dosing algorithm based on the CKD stage [12, 13].

### Modalities of renal replacement therapy

Based on the detailed description of these published reports, RRT modalities were classified into four main categories: (1) Peritoneal dialysis (PD); (2) Intermittent renal replacement therapy (IRRT) including intermittent hemodialysis (IHD), bicarbonate hemodialysis, and hemodialysis with hemoperfusion; (3) Prolonged intermittent RRT (PIRRT) including prolonged hemodialysis and sustained low efficiency dialysis (SLED); (4) Continuous renal replacement therapy (CRRT) including continuous venovenous hemofiltration (CVVH), continuous venovenous hemodialysis (CVVHD), hemofiltration (HF), continuous venovenous hemodiafiltration (CVVHDF), and high volume hemofiltration (HVHF). The intensification of RRT was defined as a modality transition from IRRT to PIRRT or CRRT, or a higher dialysis dose delivery during the same RRT modality.

### Other variables

Metformin usage was categorized into three groups: regular, suicidal and accidental. The daily and total metformin dosage was recorded, respectively, in patients with regular metformin use and suicidal or accidental ingestion. Medications that may impair kidney function and predispose the development of MALA, including nonsteroidal anti-inflammatory drugs (NSAIDs), angiotensin-converting-enzyme inhibitors (ACEIs), angiotensin II receptor blockers (ARBs), diuretics, aspirin, and statins were abstracted. Both baseline and trough estimated glomerular filtration rates (eGFR) were calculated by using the CKD Epidemiology Collaboration (CKD-EPI) equation [14].

### Statistical methods

Continuous variables were expressed as median with interquartile range and categorical variables were presented as frequencies and percentages. The Kolmogorov-Smirnov test was used together with histograms to assess the normality of continuous data. To examine the time trend of acute renal care, we stratified the study data before and after 2005 as the concepts of chronic kidney disease and acute kidney injury has rapidly evolved in the past 10 years [15, 16]. Statistical differences between parametric continuous variable were estimated by Wilcoxon signed rank test whereas differences between categorical variables were evaluated with Chi-square test.

The relationships between serum lactate concentration and serum metformin concentration and between the estimated metformin ingestion dose and serum metformin concentration were described using both parametric and nonparametric methods. Multiple logistic regression modeling was used to examine the predictors of mortality. The proposed adjustment variables included blood lactate, age, sex, RRT modality, metformin usage, and peak creatinine level. We also conducted several sensitivity analyses including (1) restricting the analyses only to cases whose metformin levels were available, (2) additional adjustment for baseline and trough eGFR, or (3) additional adjustment for appropriateness of metformin dosing (Inapproriateness vs. appropriateness) in the multiple logistic model shown in Table 3. The results were consistent (data not shown).

Two-tailed values of *P* < 0.05 were considered significant. Statistical analysis was performed using Stata SE Version 12 (StataCorp, College Station, Texas, USA) and R, version 3.0.2 (R Foundation for Statistical Computing, Vienna, Austria [www.r-project.org]). The map figure (Additional file 1: Figure S3) was drawn with the support of the googleVis library [17].

## Results

### Clinical characteristics of selected cases

For years 1977–2014, 142 case reports and 3 case series from 30 countries were identified with a total of 253 unique cases (Additional file 1: Table S2 and Figure S3) and 95.7% met the conventional diagnostic criteria of MALA. The cumulative mortality was 16.2% (41 deaths). There was no statistical difference in age at event, diabetes status, the use of metformin (including the estimated dosage), and the use of predisposing medication and other anti-diabetic agents between survival and non-survival groups (Table 1). Both groups presented with similar symptoms and signs. The level of consciousness was the most frequently reported (79.8% of all selected cases) but only 56.9% of them had acute conscious change. The least frequently recorded vital sign was body temperature (36.8%), yet 63.4% of them were classified as hypothermia (<35.5 C degree or defined by authors of the primary study) (Table 1). We found higher frequency of respiratory failure requiring endotracheal intubation (83.3%) in non-survival group than in survival group (55.1%) (*p*-value <0.01).

**Table 1:** Characteristics of published cases between 1977 and 2014 by survival status^c^

Variable	Total	Alive	Deceased	*p*-value
	[N, proportion of total study population, 253 patients]	[n, proportion of total N] 212 patients	[n, proportion of total N] 41 patients	
Demographics				
Age (yr)	64 (52.5–72.5) [252, 99.6%]	64 (54–72.5) [212, 84.1%]	60.5 (42.5–73) [40, 15.9%]	0.40
Sex (male)	41.8% [249, 98.4%]	38.5% [208, 83.5%]	58.5% [41, 16.5%]	0.02
Diabetes	91.3% [231, 96.9%]	91.0% [210, 83.0%]	93.0% [43, 17.0%]	0.76
Medical history				
Metformin use	[232, 91.7%]	[196, 84.5%]	[36, 15.5%]	0.11
Regular medication	76.7%	79.1%	63.9%	
Suicidal	22.8%	20.4%	36.1%	
Accidental	0.4%	0.5%	0.0%	
Estimated metformin dosage at MALA^a^ (g)	2.6 (1.7–5.1) [181, 72.0%]	2.6 (1.7–4.0) [154, 85.0%]	2.6 (1.0–24.5) [27, 14.9%]	0.79
Predisposing medication^b^	97.7% [83, 32.8%]	97.4% [76, 91.6%]	100% [7, 8.2%]	0.67
Other anti-diabetic agents	95.7% [47, 18.6%]	97.5% [40, 85.1%]	85.7% [7, 14.9%]	0.15
Appropriateness of metformin use				
Creatinine criteria	28.8% [80, 31.6%]	25.0% [68, 85%]	50.0% [12, 15%]	0.08
eGFR-dosing criteria	57.5% [73, 28.9%]	59.0% [61, 83.6%]	50.0% [12, 16.4%]	0.56
Symptoms & Signs				
Hypothermia	63.4% [93, 36.8%]	64.5% [76, 81.7%]	58.8% [17, 18.3%]	0.66
Hypotension	50.0% [162, 64.0%]	49.3% [136, 84.0%]	53.9% [26, 16.1%]	0.67
Abdominal pain	35.6% [149, 59.0%]	37.3% [134. 89.9%]	20.0% [15, 10.1%]	0.18
Upper GI discomfort	77.4% [155, 61.3%]	78.4% [139, 90.0%]	68.8% [16, 10.3%]	0.38
Lower GI discomfort	43.7% [151, 59.7%]	44.1% [136, 90.1%]	40.0% [15, 9.9%]	0.76
Conscious disturbance	56.9% [202, 79.8%]	56.3% [174, 86.1%]	60.7% [28, 13.9%]	0.66
Severity				
Respiratory failure	60.1% [168, 66.4%]	55.1% [138, 82.1%]	83.3% [30, 17.9%]	<0.01
Kidney Injury Profile	[203, 80.2%]	[175, 86.2%]	[28, 13.8%]	0.66
Acute kidney injury	94.6%	94.9%	92.9%	
ESRD	5.4%	5.1%	7.1%	
Biochemical profile				
Baseline creatinine (mg/dL)	1.2 (1.0–1.5) [104, 41.1%]	1.2 (0.9–1.5) [88, 84.6%]	1.3 (1.1–1.8) [16, 15.4%]	0.26
Baseline eGFR (ml/min/1.73m^2^)	50.0 (38.7–71.5) [102, 40.3%]	52.0 (39.6–71.5) [86, 84.3%]	43.5 (37.4–73.4) [16, 15.7%]	0.56
Peak creatinine (mg/dL)	7.0 (3.3–9.5) [211, 83.4%]	7.2 (4.2–9.7) [183, 86.7%]	3.2 (1.8–7.2) [28, 13.3%]	<0.01
Trough eGFR (ml/min/1.73m^2^)	6.1 (4.5–16.4) [207, 81.8%]	5.6 (4.3–11.2) [179, 86.4%]	19.2 (6.1–27.4) [28, 13.5%]	<0.01
Metformin (mg/L)	45 (27.0–74.4) [85, 33.6%]	43.9 (27.0–71.0) [71, 83.5%]	58 (16–150) [14, 16.5%]	0.61
Lactate (mmol/L)	18.0 (12.7–23) [234, 92.5%]	17 (12.5–21.2) [198, 84.6%]	22 (14.7–28.4) [36, 15.4%]	<0.01
Bicarbonate (mmol/L)	5.0 (3.1–8.0) [170, 67.1%]	5.0 (3.0–8.0) [146, 85.9%]	5.6 (4.2–8.5) [24, 14.1%]	0.19
pH	6.9 (6.8–7.1) [241, 95.3%]	6.9 (6.8–7.1) [203, 84.2%]	6.9 (6.8–7.1) [38, 15.8%]	0.56
Glucose (mg/dL)	120.9 (36–22 [118, 46.6%]	105.4 (34.3–219.5) [105, 88.1%]	198.5 (72–270) [14, 11.9%]	0.22
MALA criteria	95.7% [253, 100%]	96.2% [212, 83.8%]	92.7% [41, 16.2%]	0.31
Renal replacement therapy (RRT) profile				
Type of RRT	[251, 99.2%]	[210, 83.7%]	[41, 16.3%]	0.03
IRRT	33.9%	36.7%	19.5%	
PIRRT	13.2%	13.3%	12.2%	
CRRT	50.6%	48.6%	61.0%	
PD	2.3%	1.4%	7.3%	
RRT dose intensification	11.2% [214, 84.6%]	9.8% [184, 86.0%]	20.0% [30, 14.0%]	0.10
RRT duration (day)	2.0 (1.0–3.0) [162, 64.0%]	2.0 (1.0–4.0) [140, 86.4%]	1.5 (1.0–2.0) [22, 13.6%]	0.11
Post-event ESRD	3.9% [156, 61.7%]	3.3% [154, 98.7%]	50.0% [2, 1.3%]	<0.01

*Abbreviations*: *CRRT* continuous renal replacement therapy, *eGFR* estimated glomerular filtration rate, *IRRT* intermittent renal replacement therapy, *MALA* metformin-associated lactic acidosis, *PD* peritoneal dialysis, *PIRRT* prolonged intermittent renal replacement therapy

^a^Daily metformin dosage (g/day) for patient on regular metformin; total dosage (g) for patients with suicidal or accidental metformin exposure

^b^Medication that may predispose the development of MALA included nonsteroidal anti-inflammatory drugs, angiotensin-converting-enzyme inhibitors, angiotensin II receptor blockers, diuretics, aspirin, and statins

^c^Characteristics of MALA patients are given as percentage in each categorical variable (e.g., male) or median (interquartile range) (e.g., age) in each continuous variable; *p*-values for categorical and continuous variables are derived from Pearson Chi-squared and Wilcoxon signed rank test, respectively

### Kidney response to MALA

**Fig. 1:**
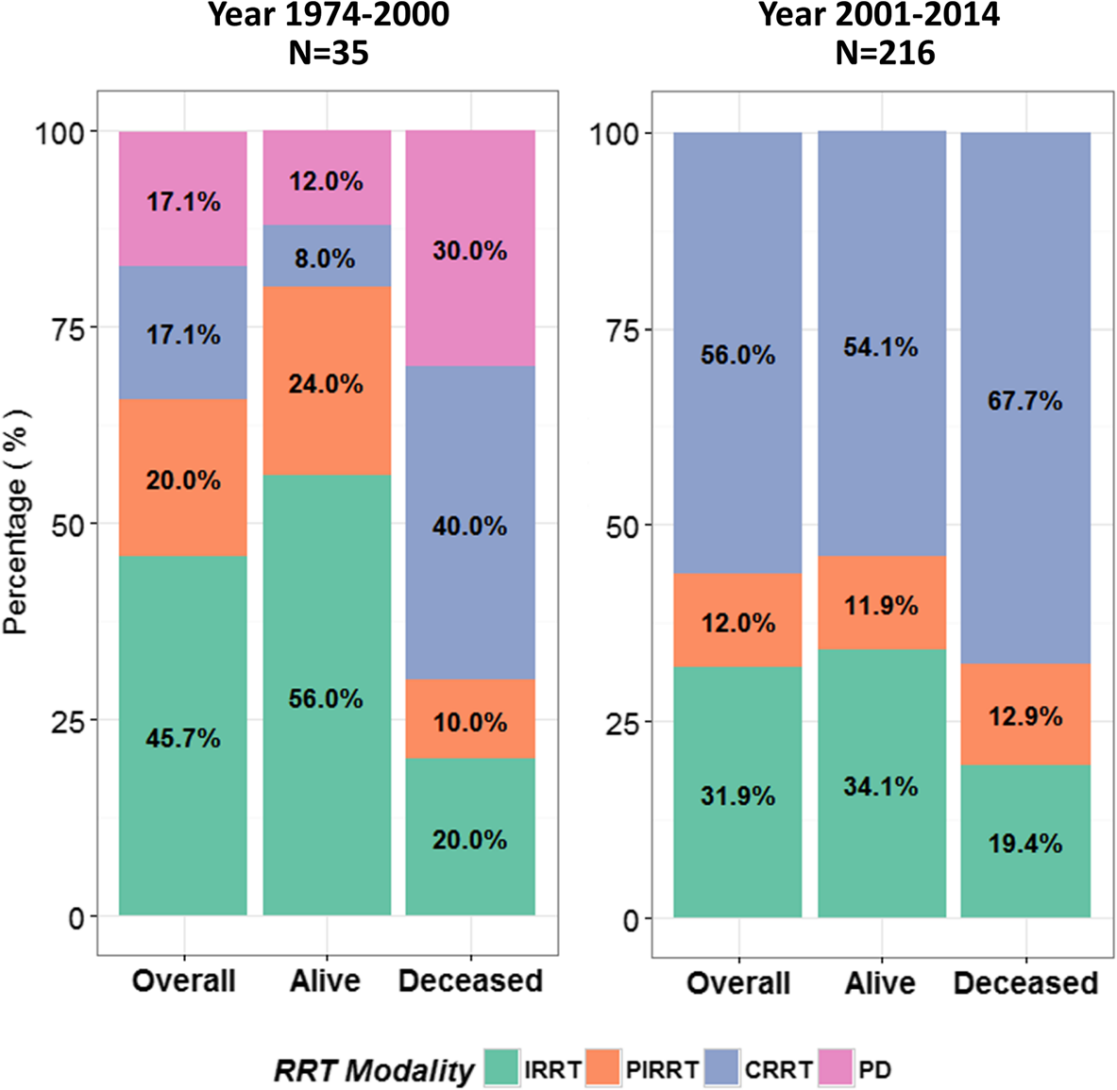
The distribution of modality of renal replacement therapy (RRT) of the enrolled cases published from 1974 to 2000 and from 2001 to 2014 by survival status. Abbreviations: CRRT, continuous renal replacement therapy; IRRT, intermittent renal replacement therapy; PD, peritoneal dialysis; PIRRT, prolonged intermittent renal replacement therapy; RRT, renal replacement therapy

Nearly 5.4% of the selected cases had end stage-renal disease requiring renal replacement therapy before the event whereas 94.6% presented with acute kidney injury (Table 1). Baseline serum creatinine level were comparable between survivors and non-survivors; however, survivors had median peaked creatinine level two times higher than non-survivors (7.2 vs. 3.2 mg/dL, *p*-value <0.01). Regarding metabolic markers of lactic acidosis, serum lactate level was significantly higher in non-survivors (median 22.0 mmol/L) than in survivors (17.0 mmol/L, *p*-value <0.01) (Table 1). We found no statistical difference in pH, blood metformin, and bicarbonate levels between the two groups. The distributions of renal replacement modalities, attempts of treatment intensification, and treatment duration between the survival and non-survival groups were no different (Table 1). After 1993, PD was no longer reported as the RRT for patients with MALA who required renal support (Fig. 1).

### Appropriateness of metformin use

Among patients receiving regular metformin dose, the proportion of inappropriate prescription of metformin was 28.9% and 57.5% based on FDA warning and a recently proposed eGFR-dosing algorithm, respectively (Table 2) [12, 13]. Patients with inappropriate metformin dosing had significantly lower baseline renal function but trough eGFR was comparable between the two groups regardless the classification criteria. When using the criteria according to FDA warning, patients with inappropriate metformin prescription were more likely to experience mortality and received a lower dose of metformin with a concomitantly lower serum lactate level compared to those of patients with appropriate dosing (Table 2). When using the eGFR-dosing approach, patients with inappropriate prescription were found to receive a significant higher dose of metformin (2550 mg vs. 1700 mg, *p*-value < 0.01) than the appropriate dosing group. However, there was no statistical difference in mortality proportion, serum metformin concentration, and serum lactate level between the two groups (Table 2).

**Table 2:** Clinical characteristics of published cases with available baseline serum creatinine and metformin dosage data stratified by dosing appropriateness^a^

	Appropriate dosing	Inappropriate dosing	*p*-value
FDA black-box warning			
n	57	23	
Age (yr)	67 (60–74)	73 (63–76)	0.28
Male (Frequency, %)	20 (35.1)	9 (39.1)	0.73
Baseline eGFR (ml/min/1.73m^2^)	58.1 (46.5–71.5)	31.2 (18.8–35.3)	<0.01
Baseline CKD stage			
CKD stage 1–2 (Frequency, %)	26 (45.6)	0 (0)	<0.01
CKD stage 3 (Frequency, %)	31 (54.4)	13 (56.5)	
CKD stage 4–5 (Frequency, %)	0 (0)	10 (43.5)	
Trough eGFR (ml/min/1.73m^2^)	*n* = 56 5.5 (4.3–8.7)	*n* = 21 6.7 (4.5–14.8)	0.66
Mortality (Frequency, %)	6 (10.5%)	6 (26.1%)	0.08
Metformin dose (mg/day)	2000 (1500–3000)	1700 (1500–2550)	0.04
Metformin concentration (mg/L)	*n* = 29 37 (22–52)	*n* = 11 26 (16–31.5)	0.21
Lactate (mmol/L)	19 (12.5–23.5)	13.4 (9.2–16.6)	0.02
eGFR-dosing algorithm			
n	31	42	
Age (yr)	67 (60–73)	70.5 (63–76)	0.46
Male (Frequency, %)	16 (51.6)	11 (26.2)	0.03
Baseline eGFR (ml/min/1.73m^2^)	65.7 (51.3–78.1)	38.2 (31.1–46.4)	<0.01
Baseline CKD stage			
CKD stage 1–2 (Frequency, %)	20 (64.5)	3 (7.1)	<0.01
CKD stage 3 (Frequency, %)	11 (35.5)	29 (69.1)	
CKD stage 4–5 (Frequency, %)	0 (0)	10 (23.8)	
Trough eGFR (ml/min/1.73m^2^)	*n* = 31 5.7 (4.5–20.5)	*n* = 40 5.5 (3.8–10.3)	0.22
Mortality (Frequency, %)	6 (19.4%)	6 (14.3%)	0.56
Metformin dose (mg/day)	1700 (1500–2000)	2550 (1700–3000)	<0.01
Metformin concentration (mg/L)	*n* = 16 41.5 (19–62.5)	*n* = 19 27 (20–43.9)	0.27
Lactate (mmol/L)	*n* = 30 17.5 (11.8–24)	*n* = 41 14.2 (11.7–21)	0.27

*Abbreviations*: *CKD* chronic kidney disease, *eGFR* estimated glomerular filtration rate, *FDA* Food and Drug Administration

^a^Characteristics are given as percentage in each categorical variable (e.g., male) or median (interquartile range) (e.g., age) in each continuous variable; *p*-values for categorical and continuous variables are derived from Pearson Chi-squared and Wilcoxon signed rank test, respectively

### Association among serum lactate level, blood metformin concentration, and mortality

**Fig. 2:**
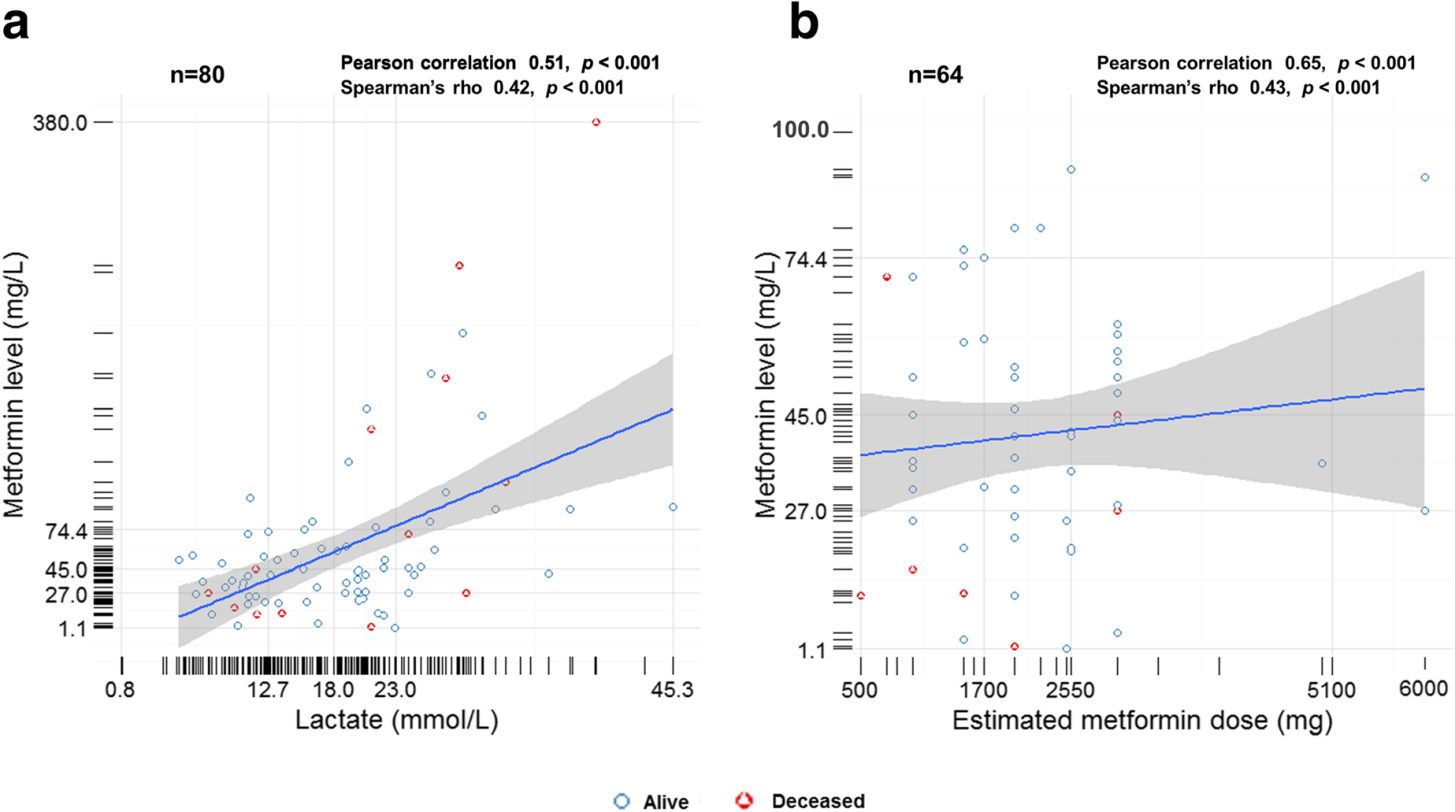
Levels of serum metformin are plotted versus the serum lactate levels (**a**) and the estimated metformin dosage (**b**). There are significant positive correlation between serum metformin and lactate levels and between serum metformin levels and the estimated metformin dosage

**Fig. 3:**
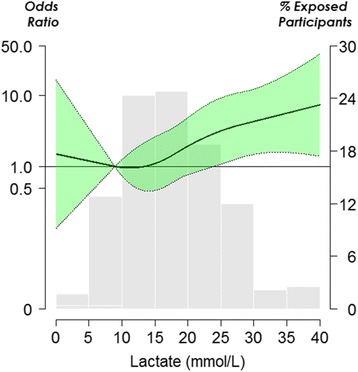
Odds ratio for metformin-associated lactic acidosis (MALA) mortality by serum lactate level in this systematic review. *Solid lines* represent adjusted odds ratios based on restricted quadratic splines for the serum lactate level, with knots at the 10th, 50th, and 90th percentiles. The *shaded green region* represent upper and lower 95% CIs. The reference value was set at 10th percentile. Adjustment factors were the same as those in Table 3. The bars represent a histogram of serum lactate distribution among enrolled cases (the extreme tails of the histogram were truncated)

Serum lactate level was positively correlated with blood metformin concentration (Fig. 2a). Blood metformin concentration was also positively related to the metformin dose (Fig. 2b). For each increase in one unit of serum lactate, there was an associated 9% (95% CI, 2–17, *p*-value = 0.01) increase in MALA-related mortality in multiple logistic regression. Factors associated with lower mortality were female gender and peaked serum creatinine level (Table 3). In the dose-response analysis, we found a linear association between serum lactate level and MALA-related mortality, particularly in the range of greater than 20 mmol/L (Fig. 3).

**Table 3:** Odds ratios (ORs) with 95% of confidence interval (CI) of metformin-associated lactic acidosis (MALA)-related mortality for potentially predictive factors by univariable and multiple logistic regression

Variable	Deceased/survivor (total)	Crude analysis OR (95% CI)	*p*-value	Multiple-adjustment OR (95% CI) (deceased/survivor: 23/152, total = 175)	*p*-value
Blood lactate (mmol/L)	36/198 (234)	1.08 (1.03, 1.13)	0.001	1.09 (1.02, 1.17)	0.009
Age (yr)	40/212 (252)	0.99 (0.97, 1.01)	0.297	1.03 (0.98, 1.08)	0.236
Sex (ref: male)	41/208 (249)	0.44 (0.22, 0.87)	0.019	0.22 (0.08, 0.67)	0.007
RRT modality	41/210 (251)				
IRRT		Ref	—	Ref	—
PIRRT		1.72 (0.52, 5.70)	0.376	1.28 (0.15, 10.7)	0.818
CRRT		2.36 (1.01, 5.52)	0.048	3.13 (0.81, 12.1)	0.098
PD		9.63 (1.67, 55.8)	0.012	3.11 (0.10, 96.1)	0.517
Metformin usage	36/196 (232)				
Suicidal (Ref: regular use)		2.19 (1.02, 4.70)	0.044	0.80 (0.15, 4.23)	0.790
Peak creatinine level (mg/dl)	28/183 (211)	0.80 (0.70, 0.91)	0.001	0.77 (0.65, 0.91)	0.003

*Abbreviations*: *CRRT* continuous renal replacement therapy, *IRRT* intermittent renal replacement therapy, *PD* peritoneal dialysis, *PIRRT* prolonged intermittent renal replacement therapy, *RRT* renal replacement therapy

## Discussion

This systematic review suggests that the cumulative mortality of MALA with kidney failure in case reports or case series published between 1977 and 2014 with biochemical data available at individual level is 17.2%, which is much lower than previously documented high mortality rates to about 50% based on single center experience [18]. We also found serum lactate level is a significant predictor for MALA-related mortality with a positive linear relationship particularly at greater than 20 mmol/L. From a treatment planning perspective, renal replacement therapy (RRT) may play a critical role in reversing disturbances of lactic homeostasis but no single RRT modality shows superior performance than the other. Although our study contributes novel observations regarding MALA with kidney failure, the results need to be interpreted with caution due to publication bias, heterogeneity between studies, and moderate sample size.

The observed positive association between inappropriate dosing defined by conventional FDA serum creatinine criteria and mortality among MALA patients requiring RRT suggested the iatrogenic and preventable nature of MALA [12]. More interestingly, this association was inverted if the inappropriateness was defined by a recently proposed dosing recommendation (eGFR-dosing algorithm) [13]. Thus, the increasing sensitivity of alarming inappropriateness gained by applying the eGFR-dosing algorithm was of questionable clinical significance in the context of risk prevention. Furthermore, most cases with classification disagreement between the two different definitions were patients with CKD stage 3. This finding indirectly supports that a cautious expansion of metformin use in patients with CKD stage 3 or 4 may be appropriate and the optimal dosing requires more evidence to justify its long term efficacy and safety profile among CKD population [9]. The drastic decline of kidney function among patients with appropriate metformin dose with concomitantly higher metformin and lactate concentration implied the primary etiologies of AKI played an important role in the development of MALA.

The traditional target of metformin is inhibition of mitochondrial complex-1, which subsequently impair the efficiency of mitochondrial oxidative phosphorylation and Adenosine triphosphate (ATP) production [19]. Mechanistic studies have linked metformin to activation of adenosine monophosphate (AMP)-activated protein kinase (AMPK) or inhibition of cyclic AMP formation following the drop of cellular energy charge [20, 21]. A recent study demonstrated that metformin inhibits mitochondrial glycerophosphate dehydrogenase (mGPD) causing an increased cytosolic redox state (increased cytosolic NADH/NAD^+^ ratio) that hinder the conversion of lactate to pyruvate by lactate dehydrogenase [22]. When serum metformin level increases abnormally due to either ingestion of a large amount of metformin and/or to decreased kidney clearance of metformin, excessive production of lactate and proton will ensue.

**Fig. 4:**
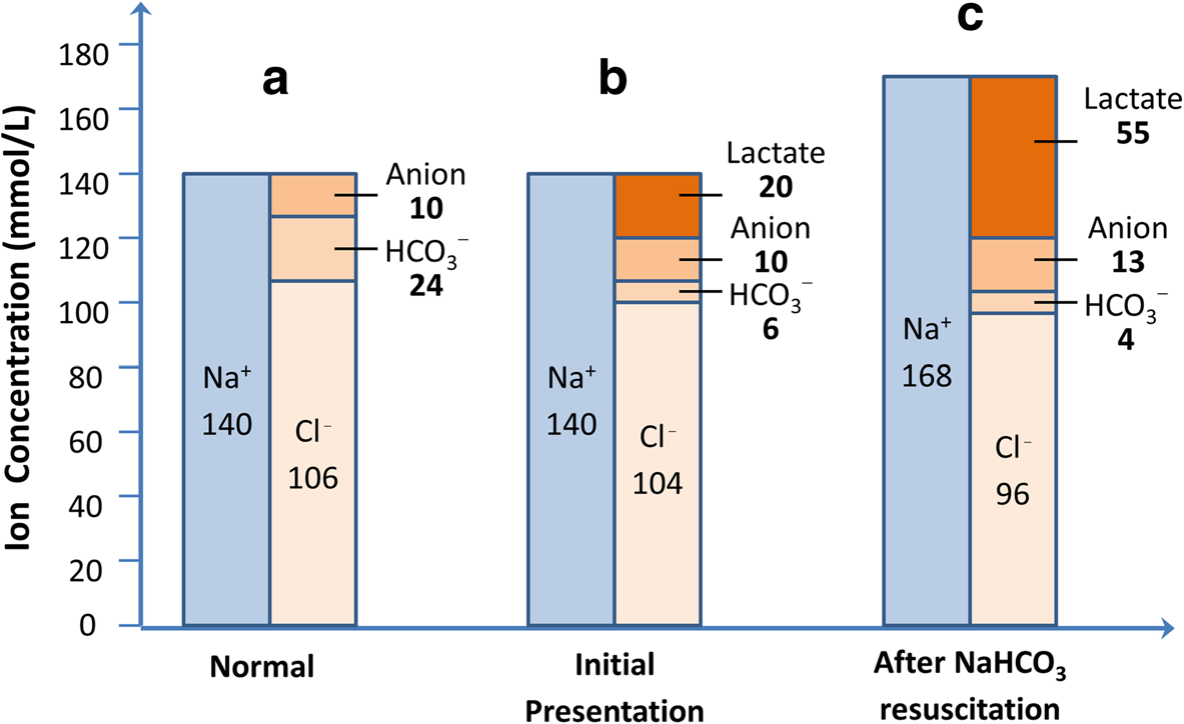
The extracellular ionic compositional progression by Gamblegrams from our MALA case (The detailed information of the case was described in the Additional file 1: Figure S4). **a** normal physiology composition of major ions in extracellular fluid (ECF). **b** A 50-year-old woman case initially presented with severe lactic acidosis a few hours after suicidal metformin ingestion. Intracellular lactate was transported along with equivalent amount of protons into ECF via monocarboxylate transporters. Excess ECF protons were then buffered by bicarbonate and turned into H_2_O and CO_2_. Consequently, the increased amounts of lactate were approximately equivalent to the decreased consumption of bicarbonate. However, the failing kidney function could not replenish the consumed bicarbonate through renal ammoniagenesis or enhanced reabsorption, leading to increasingly severe acidosis. **c** To reverse acidosis, 622.5 mmol of sodium bicarbonate was infused to neutralize protons moving out of the cell to ECF, along with lactate. The iatrogenic hypernatremia to 168 mmol/L created an extra anion space that allowed more lactate to move from intracellular fluid to ECF, resulting in more significant hyperlactatemia

Cumulating intracellular lactate is then transported into extracellular fluid (ECF) concomitantly with hydrogen 1:1 by proton-coupled monocarboxylate transporters (MCTs) [23]. To maintain acid-base homeostasis in ECF, proton reacts with bicarbonate to form water and carbon dioxide whereas lactate is metabolized by gluconeogenesis or oxidation after converting to pyruvate. Bicarbonate is then replenished by kidney via tubular reabsorption or net acid secretion [24, 25]. This homeostasis is energy dependent and driven by decreased NADH/NAD^+^ ratio at cytosol, which is highly vulnerable in the context of MALA featuring with energy failure and increased intracellular redox status. As a result, the rapidly accumulating extracellular lactate and proton will consume equivalent amounts of bicarbonate in ECF until the maximum tolerable lactate threshold, around 20 mmol/L (Fig. 4b), is reached. Beyond this intrinsic threshold, the export of intracellular lactate/proton is then severely limited, followed by irreversible cellular damage. However, among patients suffering from severe volume contraction and pre-existing metabolic alkalosis, the endogenous bicarbonate in ECF prior to the development MALA may raise this lactate threshold beyond 20 mmol/L [26]. To reverse the severe uncompensated acidosis, exogenous sodium bicarbonate infusion not only raises serum bicarbonate to neutralize proton acidity but also causes iatrogenic hypernatremia to create an extra “anion space” for excessive lactate in the price of systemic hyperosmolality (Fig. 4c). In this scenario, timely renal replacement therapy is the therapy of choice to remove excessive metformin and to correct intracellular acidosis while maintaining osmolar homeostasis as exemplified in a patient with MALA and an extremely high serum lactate level of 55 mmol/L successfully treated at our hospital (Additional file 1: Figure S4). This case highlighted the dynamics of how prescription of RRT was tailored to meet incredibly high metabolic demand.

Our findings are well consistent with a recent comprehensive review by Calello et al., in which a decision making algorithm for RRT in MALA was proposed [27]. The decision to initiate RRT is mainly triggered by serum lactate level and refractory severe acidosis. The proposed cut-off value of lactate, 20 mmol/L, coincides with our dose-response analysis indicating a linear increase in mortality, particularly at lactate concentrations greater than 20 mmol/L [27]. The role of serum lactate in metformin-related acidosis remains controversial from the diagnostic perspective as the elevation of lactate are usually induced from mutually reinforcing factors, for instance, predisposing impaired kidney function and resultant acidemia-related circulatory failure. But reversely, serum lactate could serve as a useful integrated marker to guide the initiation and adjustment of RRT. Compared to the general intensive care unit (ICU) population in which baseline serum lactate level is a reliable predictor of ICU mortality, the proposed lactate threshold for an increased mortality risk, 20 mmol/L, is much higher than the usual alarming lactate range of 2.0–4.0 mmol/L in patient’s lactemia mainly due to hypo-perfusion (e.g., sepsis and trauma) [28]. Such discrepancy may reflect the unique pathogenesis of MALA – cellular energy failure and impaired redox state [21, 22]. More than half of the study population had hypothermia on arrival at the care hospital. This provides indirect evidence of cellular energy failure, as thermogenesis driven by ATP turnover would be compromised in MALA [29]. In the era of practicing individualized medicine, the bottom-line is that the modality of RRT should match to the patient’s hemodynamic stability and the dosing of RRT has to properly correct acidosis and avoid iatrogenic hypernatremia, and should be adjusted to meet patient’s metabolic demand (exemplified in Additional file 1: Figure S4). To address this current knowledge gap regarding the RRT prescription in MALA patients with severe acute kidney injury (e.g., Acute Kidney Injury Network (AKIN) stage 3) [16], an international consortium is needed to prospectively enroll patients worldwide using a standardized case report form to record detailed information about the metformin toxicokinetics and the three domains of RRT including mode, dose, and timing.

There were several interesting but unexpected findings in this study. First, a higher peak level of serum creatinine was associated with favorable survival. Indeed, apparent renal failure may help clinicians generate a high index of suspicion for MALA with appropriate evaluation resulting in earlier diagnosis and expedient delivery of RRT. On the other hand, patients with higher serum creatinine level at the initial presentation may also have a relatively indolent disease course rather than an acute episode of fulminant metabolic acidosis and circulatory collapse. However, our data was not sufficient to determine whether the timing of RRT was early or late as it is difficult, if not impossible, to define the onset of MALA. Second, female patients had a better survival than male patients. Although it is likely that the efficiency of RRT may be better among female patients due to smaller body size, the information of body mass index (BMI) was rarely available in the published reports. Future prospective research is warranted to clarify the sex difference or the effect of BMI.

### Limitation

Although the comprehensive nature of this systematic review can be interpreted as strength, several limitations deserve particular mention in the current work. Publication bias is a critical concern in this study as severe cases with unexpected favorable outcomes may be more likely reported by clinicians and accepted by medical journals. We compared the mortality rate of selected MALA patients between 1995 and 2010 with those from a pharmacovigilence database covering the same time span and the results were not too different (17.7% vs. 25%) [30]. The much lower mortality in our study population compared to the historical series may reflect the efficient support role of the RRT in maintaining the acid-base homeostasis in MALA [18, 31]. On the other hand, misclassification of the diagnosis of MALA can not be completely excluded. However, as all enrolled reports were peer-reviewed, misdiagnosis of MALA may be minimized. Residual confounding, especially unknown or unmeasured confounding, is another serious concern in this study. For example, it is not possible to standardize the onset and severity of MALA among the retrospectively enrolled cases to determine whether the intervention of RRT is early or late. Subsequently, the conclusions drawn from this study regarding the determinants of mortality are very limited to infer causality and generalizability [32]. However, this real-world issue may not be completely avoided by prospective studies. Considering the rarity and severity of MALA, systematically analyzing case reports in the literature may be a realistic approach to describe the practice pattern and trend of RRT use in MALA.

## Conclusions

Our study suggests that predialysis level of serum lactate level is an important marker of mortality in MALA patients requiring RRT with a linear dose-response relationship. There is no differential survival advantage among RRT modalities and significant knowledge gaps remain regarding the optimal timing, mode, and dose of RRT. Standardization of MALA case report form would be the fundamental step toward guideline development.

## Additional file

Additional file 1: Supplementary tables and figures. **Table S1.** Database search strategies for RRT and MALA. **Table S2.** Case reports presented patients with MALA requiring RRT between 1974 and 2014. Studies were ordered by year of publication and by name of author within each year. **Figure S1.** Flow diagram of study selection process. **Figure S2.** The diagram of the definitions and coding rules of key variables. **Figure S3.** The distribution of number of published case reports by countries. **Figure S4.** The detailed summary of disease course of a 50-year-old diabetic woman with severe MALA after suicidal metformin ingestion of more than 40 g treated at China Medical University Hospital in Taiwan. The initial investigation at local hospital revealed severe metabolic acidosis and a total of 622.5 mmol sodium bicarbonate was administered ﻿﻿﻿before transferring﻿﻿ ﻿to our hospital. An 8-h SLED-f was immediately conducted for her hypernatremia (Na: 168 mmol/L), anuric kidney injury (serum creatinine 2.05 mg/dL), severe metabolic acidosis (pH: 6.84 and HCO3^−^: 4.4 mmol/L), and an extremely high serum lactate level to 55.5 mmol/L. The RRT modality was further switched to HVHF for refractory shock and lactic acidosis. Both SLED-f and HVHF were sequentially performed again in the first 48 h . Although the total amount of sodium bicarbonate supplementation reached astronomically high levels (1660 mmol via intravenous infusion; 4400 mmol via replacement fluid), the serum sodium concentration could be maintained at normal range within 24 h of RRT initiation. The RRT modalities were shifted to CVVH and then IHD as condition stabilized, and the patient obtained independence from RRT after 20 days of treatment. Her peak serum lactate level of 55.5 mmol/l is more than any case we have reviewed in this systematic review. Abbreviations: Bicarb, each yellow rectangle of “Bicarb” stands for 250 ml of 7% sodium bicarbonate; BT, body temperature; CVVH, continuous venovenous hemofiltration (replacement flow = 35 ml/kg/h); HR, heart rate; HVHF, high volume hemofiltration (replacement flow = 70 ml/kg/h); IE, inotropic equivalents; SBP, systolic blood pressure; SLED-f, sustained low-efficiency hemodiafiltration. (DOCX 768 kb)

## Abbreviations

ACEIs: Angiotensin-converting-enzyme inhibitors
AKI: Acute kidney injury
AMP: Adenosine monophosphate
AMPK: Adenosine monophosphate-activated protein kinase
ARBs: Angiotensin II receptor blockers
ATP: Adenosine triphosphate
CRRT: Continuous renal replacement therapy
CVVH: Continuous venovenous hemofiltration
CVVHD: Continuous venovenous hemodialysis
CVVHDF: Continuous venovenous hemodiafiltration
ECF: Extracellular fluid
ESRD: End stage renal disease
FDA: Food and Drug Administration
HF: Hemofiltration
HVHF: High volume hemofiltration
IHD: Intermittent hemodialysis
IRRT: Intermittent renal replacement therapy
MALA: Metformin-associated lactic acidosis
MCTs: Monocarboxylate transporters
mGPD: Mitochondrial glycerophosphate dehydrogenase
NAD: Nicotinamide adenine dinucleotide
NSAID: Nonsteroidal anti-inflammatory drugs
PD: Peritoneal dialysis
PIRRT: Prolonged intermittent renal replacement therapy
RRT: Renal replacement therapy
