# Supplementary material for: Serum lactate level and mortality in metformin-associated lactic acidosis requiring renal replacement therapy: a systematic review of case reports and case series
Source: BMC Nephrol. 2017 Jul 10;18:229. doi: 10.1186/s12882-017-0640-4 (PMC5504722; doi:10.1186/s12882-017-0640-4)
Supplement: Additional file 1: — Supplementary tables and figures. Table S1. Database search strategies for RRT and MALA. Table S2. Case reports presented patients with MALA requiring RRT between 1974 and 2014. Studies were ordered by year of publication and by name of author within each year. Figure S1. Flow diagram of study selection process. Figure S2. The diagram of the definitions and coding rules of key variables. Figure S3. The distribution of number of published case reports by countries. Figure S4. The detailed summary of disease course of a 50-year-old diabetic woman with severe MALA after suicidal metformin ingestion of more than 40 g treated at China Medical University Hospital in Taiwan. The initial investigation at local hospital revealed severe metabolic acidosis and a total of 622.5 mmol sodium bicarbonate was administered ﻿﻿﻿before transferring﻿﻿ ﻿to our hospital. An 8-h SLED-f was immediately conducted for her hypernatremia (Na: 168 mmol/L), anuric kidney injury (serum creatinine 2.05 mg/dL), severe metabolic acidosis (pH: 6.84 and HCO3−: 4.4 mmol/L), and an extremely high serum lactate level to 55.5 mmol/L. The RRT modality was further switched to HVHF for refractory shock and lactic acidosis. Both SLED-f and HVHF were sequentially performed again in the first 48 h . Although the total amount of sodium bicarbonate supplementation reached astronomically high levels (1660 mmol via intravenous infusion; 4400 mmol via replacement fluid), the serum sodium concentration could be maintained at normal range within 24 h of RRT initiation. The RRT modalities were shifted to CVVH and then IHD as condition stabilized, and the patient obtained independence from RRT after 20 days of treatment. Her peak serum lactate level of 55.5 mmol/l is more than any case we have reviewed in this systematic review. Abbreviations: Bicarb, each yellow rectangle of “Bicarb” stands for 250 ml of 7% sodium bicarbonate; BT, body temperature; CVVH, continuous venovenous hemofiltration (replacement flow = 35 ml/kg/ [file 12882_2017_640_MOESM1_ESM.docx]

**Table S1.** Database search strategies for renal replacement therapy and metformin-associated lactic acidosis (MALA).

| **Date** | **Sep 15, 2014** |
| --- | --- |
| **Strategy** | We combined the results for renal replacement therapy search strategy (#1 below) with the results for the metformin-related lactic acidosis (MALA) search strategy (#2 and #3 combined below) in each database |
| **Database** | **PubMed** |
| **#1. Renal replacement therapy [196,312]** | "Renal Replacement Therapy"[Mesh] OR "Dialysis"[Mesh] OR "Renal Dialysis"[Mesh] OR "Hemodialysis Units, Hospital"[Mesh] OR "Hemodiafiltration"[Mesh] OR "Hemofiltration"[Mesh] OR CRRT OR CVV* OR CAVH OR CAVHD OR CAVHDF OR IHD OR SCUF OR SLED OR SLEDD OR SLEDDF OR SLEDD-F OR “continuous renal replacement therapy” OR “Continuous veno-venous hemodialysis” OR “Continuous Veno-Venous Hemofiltration” OR “continuous arteriovenous haemofiltration” OR “Continuous arteriovenous hemodiafiltration” OR “Continuous arteriovenous hemodialysis” OR “intermittent hemodialysis” OR “Slow Continuous Ultrafiltration” OR “Sustained low-efficiency dialysis” OR “Sustained low-efficiency daily dialysis” OR “Sustained low-efficiency daily diafiltration” OR “extended daily dialysis” |
| **#2. Metformin [21,897]** | "Metformin"[Mesh] OR "metformin" OR "Biguanides"[Mesh] OR “biguanides” OR “metformin/adverse effects” |
| **#3. Lactic acidosis [6,566]** | "Acidosis, Lactic"[Mesh] OR “lactic acidosis” OR “metformin-related lactic acidosis” OR “metformin-associated lactic acidosis” OR “MALA” OR “metformin-induced lactic acidosis” OR “Acidosis, Lactic/chemically induced” |
| **Database** | **EMBASE** |
| **#1. Renal replacement therapy [178,909]** | ‘Renal Replacement Therapy’/exp OR ‘Dialysis’/exp OR ‘Renal Dialysis’/exp OR ‘Hemodialysis Units, Hospital’/exp OR ‘Hemodiafiltration’/exp OR ‘Hemofiltration’/exp OR CRRT OR CVV* OR CAVH OR CAVHD OR CAVHDF OR IHD OR SCUF OR SLED OR SLEDD OR SLEDDF OR SLEDD-F OR ‘continuous renal replacement therapy’ OR ‘Continuous veno-venous hemodialysis’ OR ‘Continuous Veno-Venous Hemofiltration’ OR ‘continuous arteriovenous haemofiltration’ OR ‘Continuous arteriovenous hemodiafiltration’ OR ‘Continuous arteriovenous hemodialysis’ OR ‘intermittent hemodialysis’ OR ‘Slow Continuous Ultrafiltration’ OR ‘Sustained low-efficiency dialysis’ OR ‘Sustained low-efficiency daily dialysis’ OR ‘Sustained low-efficiency daily diafiltration’ OR ‘extended daily dialysis’ |
| **#2. Metformin [46,745]** | ‘Metformin’/exp OR ‘metformin’ OR ‘Biguanides’/exp OR ‘biguanides’ OR ‘metformin/adverse effects’ |
| **#3. Lactic acidosis [13,602]** | ‘Acidosis, Lactic’/exp OR ‘Acidosis, Lactic’ OR ‘lactic acidosis’/exp OR ‘lactic acidosis’ OR ‘metformin-related lactic acidosis’/exp OR ‘metformin-related lactic acidosis’ OR ‘metformin-associated lactic acidosis’/exp OR 'metformin-associated lactic acidosis’ OR ‘MALA’ OR ‘metformin-induced lactic acidosis’/exp OR ‘metformin-induced lactic acidosis’ OR ‘Acidosis, Lactic/chemically induced’ |

**Table S2.** Case reports presented patients with metformin-associated lactic acidosis requiring renal replacement therapy between 1974 and 2014. Studies were ordered by year of publication and by name of author within each year.

| **1^st^ Author, Reference** | **Year** | **Country** | **Study sample size** | **Age range** | **Male (%)** |
| --- | --- | --- | --- | --- | --- |
| Hayat[[1](#_ENREF_1)] | 1974 | France | 2 | 56-74 | 50 |
| Bismuth[[2](#_ENREF_2)] | 1976 | France | 1 | 37 | 100 |
| Assan[[3](#_ENREF_3)] | 1977 | France | 4 | 38-67 | 75 |
| Larcan[[4](#_ENREF_4)] | 1981 | France | 2 | 53-56 | 50 |
| Chalopin[[5](#_ENREF_5)] | 1984 | France | 1 | 64 | 0 |
| Perrot [[6](#_ENREF_6)] | 1986 | France | 2 | 67-74 | 0 |
| Huguet[[7](#_ENREF_7)] | 1986 | France | 1 | 38 | 0 |
| Lalau[[8](#_ENREF_8)] | 1987 | France | 5 | 48-80 | 40 |
| Lacroix[[9](#_ENREF_9)] | 1988 | France | 1 | 61 | 0 |
| Khan[[10](#_ENREF_10)] | 1993 | UK | 1 | 76 | 0 |
| Kovacs[[11](#_ENREF_11)] | 1996 | Canada | 1 | 72 | 100 |
| Pearlman[[12](#_ENREF_12)] | 1996 | US | 1 | 60 | 100 |
| Heaney[[13](#_ENREF_13)] | 1997 | UK | 1 | 29 | 100 |
| Jurovich[[14](#_ENREF_14)] | 1997 | US | 1 | 67 | 100 |
| Mercker[[15](#_ENREF_15)] | 1997 | Germany | 1 | 66 | 100 |
| Schmidt[[16](#_ENREF_16)] | 1997 | US | 1 | 62 | 0 |
| Darwich[[17](#_ENREF_17)] | 1998 | US | 1 | 60 | 0 |
| Gainza[[18](#_ENREF_18)] | 1998 | Spain | 1 | 84 | 0 |
| Teale[[19](#_ENREF_19)] | 1998 | UK | 3 | 25-60 | 66.7 |
| Stefansson[[20](#_ENREF_20)] | 1999 | Sweden | 1 | 66 | 0 |
| Houwerzijl[[21](#_ENREF_21)] | 2000 | Netherlands | 1 | 52 | 0 |
| Reeker[[22](#_ENREF_22)] | 2000 | Germany | 1 | 62 | 0 |
| Doorenbos[[23](#_ENREF_23)] | 2001 | Netherlands | 1 | 66 | 0 |
| Ellis[[24](#_ENREF_24)] | 2001 | Canada | 1 | 71 | 0 |
| Kruse[[25](#_ENREF_25)] | 2001 | US | 1 | 76 | 0 |
| Barrueto[[26](#_ENREF_26)] | 2002 | US | 1 | 58 | 100 |
| Berner[[27](#_ENREF_27)] | 2002 | Germany | 1 | 83 | 0 |
| Chang[[28](#_ENREF_28)] | 2002 | Taiwan | 5 | 25-80 | 40 |
| Chu[[29](#_ENREF_29)] | 2003 | Taiwan | 1 | 75 | 0 |
| Gjedde[[30](#_ENREF_30)] | 2003 | Denmark | 1 | 70 | 100 |
| Heras[[31](#_ENREF_31)] | 2003 | Spain | 1 | 57 | 0 |
| Nisse[[32](#_ENREF_32)] | 2003 | France | 1 | 42 | 100 |
| Pertek[[33](#_ENREF_33)] | 2003 | France | 1 | 65 | 0 |
| Price[[34](#_ENREF_34)] | 2003 | Australia | 1 | 58 | 0 |
| Schure[[35](#_ENREF_35)] | 2003 | Netherlands | 1 | 72 | 0 |
| Iwai[[36](#_ENREF_36)] | 2004 | Japan | 1 | 38 | 100 |
| Mallick[[37](#_ENREF_37)] | 2004 | UK | 1 | 61 | 0 |
| Moerer[[38](#_ENREF_38)] | 2004 | Germany | 1 | 79 | 0 |
| Peña[[39](#_ENREF_39)] | 2004 | Spain | 1 | 70 | NA^*^ |
| Panzer[[40](#_ENREF_40)] | 2004 | Germany | 1 | 42 | 100 |
| von Mach[[41](#_ENREF_41)] | 2004 | Germany | 1 | 61 | 0 |
| Harvey[[42](#_ENREF_42)] | 2005 | UK | 1 | 14 | 0 |
| Lacher[[43](#_ENREF_43)] | 2005 | Germany | 1 | 15 | 0 |
| Runge[[44](#_ENREF_44)] | 2005 | Germany | 1 | 74 | 100 |
| Clare[[45](#_ENREF_45)] | 2006 | UK | 3 | 54-71 | 33.3 |
| Friesecke[[46](#_ENREF_46)] | 2006 | Germany | 1 | 68 | 0 |
| Gudmundsdottir[[47](#_ENREF_47)] | 2006 | Norway | 5 | 49-81 | 20 |
| Guo[[48](#_ENREF_48)] | 2006 | Canda | 2 | 37-53 | 100 |
| Aquarius[[49](#_ENREF_49)] | 2007 | Netherlands | 1 | 57 | 0 |
| EL-Hennawy[[50](#_ENREF_50)] | 2007 | US | 1 | 61 | 100 |
| Galea[[51](#_ENREF_51)] | 2007 | UK | 1 | 46 | 100 |
| González Losada[[52](#_ENREF_52)] | 2007 | Spain | 1 | 51 | 0 |
| La Maza Pereg[[53](#_ENREF_53)] | 2007 | Spain | 1 | 69 | 100 |
| Lopez[[54](#_ENREF_54)] | 2007 | Spain | 1 | 61 | 0 |
| Radej[[55](#_ENREF_55)] | 2007 | Czech | 1 | 63 | 0 |
| Shenoy[[56](#_ENREF_56)] | 2007 | US | 1 | 48 | 100 |
| Silvestre[[57](#_ENREF_57)] | 2007 | Portugal | 2 | 69-77 | 50 |
| Almirall[[58](#_ENREF_58)] | 2008 | Spain | 5 | 66-70 | 0 |
| Audia[[59](#_ENREF_59)] | 2008 | US | 1 | 50 | 0 |
| Balik[[60](#_ENREF_60)] | 2008 | Czech | 1 | 82 | 100 |
| Bruijstens[[61](#_ENREF_61)] | 2008 | Netherlands | 3 | 45-72 | 33.3 |
| Di Grande[[62](#_ENREF_62)] | 2008 | Italy | 1 | 75 | 0 |
| Khan[[63](#_ENREF_63)] | 2008 | Doha-Qatar | 1 | 45 | 0 |
| Redha[[64](#_ENREF_64)] | 2008 | Kuwait | 1 | 20 | 0 |
| Teutonico[[65](#_ENREF_65)] | 2008 | Italy | 1 | 64 | 0 |
| Abramo[[66](#_ENREF_66)] | 2009 | Italy | 1 | 42 | 0 |
| Barbani [[67](#_ENREF_67)] | 2009 | Italy | 3 | 63-77 | 66.7 |
| Brouwers[[68](#_ENREF_68)] | 2009 | Netherlands | 1 | 62 | 0 |
| Chang[[69](#_ENREF_69)] | 2009 | Taiwan | 1 | 56 | 0 |
| Dora[[70](#_ENREF_70)] | 2009 | Brazil | 1 | 52 | 100 |
| Fitzgerald[[71](#_ENREF_71)] | 2009 | US | 1 | 49 | 0 |
| Hagset[[72](#_ENREF_72)] | 2009 | Norway | 1 | 58 | 100 |
| Kreshak[[73](#_ENREF_73)] | 2009 | US | 1 | 67 | 0 |
| Leung[[74](#_ENREF_74)] | 2009 | US | 2 | 58-78 | 50 |
| Mizzi[[75](#_ENREF_75)] | 2009 | Italy | 1 | 53 | 100 |
| Pan[[76](#_ENREF_76)] | 2009 | Singapore | 1 | 74 | 100 |
| Turkcuer[[77](#_ENREF_77)] | 2009 | Turkey | 1 | 30 | 0 |
| Wen[[78](#_ENREF_78)] | 2009 | Taiwan | 9 | 48-79 | 44.4 |
| Yang[[79](#_ENREF_79)] | 2009 | Taiwan | 1 | 43 | 0 |
| zur Nieden[[80](#_ENREF_80)] | 2009 | Germany | 2 | 50-85 | 0 |
| Arroyo[[81](#_ENREF_81)] | 2010 | US | 1 | 49 | 0 |
| Carrillo[[82](#_ENREF_82)] | 2010 | Mexico | 1 | 75 | 100 |
| Dell’Aglio[[83](#_ENREF_83)] | 2010 | US | 1 | 40 | 0 |
| Gamst [[84](#_ENREF_84)] | 2010 | Denmark | 1 | 61 | 100 |
| Giuliani[[85](#_ENREF_85)] | 2010 | Italy | 1 | 47 | 100 |
| Huberlant[[86](#_ENREF_86)] | 2010 | Belgium | 1 | 71 | 0 |
| Kavalc[[87](#_ENREF_87)] | 2010 | Turkey | 1 | 55 | 100 |
| Livshits[[88](#_ENREF_88)] | 2010 | USA | 1 | 60 | 100 |
| Olivera-Gonza´lez[[89](#_ENREF_89)] | 2010 | Spain | 1 | 73 | 0 |
| Orsi[[90](#_ENREF_90)] | 2010 | USA | 1 | 75 | 0 |
| Thajeb[[91](#_ENREF_91)] | 2010 | USA | 1 | 36 | 100 |
| Walsh[[92](#_ENREF_92)] | 2010 | US | 1 | 27 | 0 |
| Akoglu[[93](#_ENREF_93)] | 2011 | Turkey | 1 | 34 | 100 |
| Aperis[[94](#_ENREF_94)] | 2011 | Greece | 1 | 74 | 100 |
| Chow[[95](#_ENREF_95)] | 2011 | USA | 1 | 55 | 100 |
| Cosenza[[96](#_ENREF_96)] | 2011 | US | 1 | 51 | 100 |
| Devetzis[[97](#_ENREF_97)] | 2011 | Greece | 2 | 61-71 | 0 |
| Gómez[[98](#_ENREF_98)] | 2011 | Spain | 1 | 81 | 0 |
| Hellervik[[99](#_ENREF_99)] | 2011 | USA | 1 | 56 | 0 |
| Jagia[[100](#_ENREF_100)] | 2011 | Kuwait | 1 | 36 | 100 |
| Keller[[101](#_ENREF_101)] | 2011 | France | 6 | 54-81 | 16.6 |
| Miller[[102](#_ENREF_102)] | 2011 | USA | 1 | 39 | 100 |
| Miller [[103](#_ENREF_103)] | 2011 | US | 1 | 49 | 100 |
| Parekh[[104](#_ENREF_104)] | 2011 | USA | 1 | 43 | 100 |
| Parke[[105](#_ENREF_105)] | 2011 | USA | 1 | 62 | 100 |
| Perrone[[106](#_ENREF_106)] | 2011 | US | 3 | 40-69 | 0 |
| Pikwer[[107](#_ENREF_107)] | 2011 | Sweden | 1 | 55 | 0 |
| Rawat[[108](#_ENREF_108)] | 2011 | USA | 1 | 60 | 100 |
| Rifkin[[109](#_ENREF_109)] | 2011 | US | 1 | 50 | 100 |
| Sencan[[110](#_ENREF_110)] | 2011 | Turkey | 3 | 20-38 | 33.3 |
| Soyoral[[111](#_ENREF_111)] | 2011 | Turkey | 2 | 17-34 | 50 |
| Surani[[112](#_ENREF_112)] | 2011 | USA | 1 | 55 | 100 |
| Yeung[[113](#_ENREF_113)] | 2011 | Hong Kong | 19 | 43-89 | 21.1 |
| Baró-Serra[[114](#_ENREF_114)] | 2012 | Spain | 7 | 52-78 | 85.7 |
| Dichtwald[[115](#_ENREF_115)] | 2012 | Isreal | 6 | 57-84 | 33.3 |
| Jorgensen[[116](#_ENREF_116)] | 2012 | Denmark | 2 | 56-61 | 0 |
| Montini[[117](#_ENREF_117)] | 2012 | France | 4 | 53-87 | 50 |
| Mujtaba[[118](#_ENREF_118)] | 2012 | US | 1 | 70 | 100 |
| Mustafa[[119](#_ENREF_119)] | 2012 | US | 1 | 54 | 0 |
| Rathnapala[[120](#_ENREF_120)] | 2012 | Sri Lanka | 1 | 18 | 0 |
| Schousboe[[121](#_ENREF_121)] | 2012 | Denmark | 4 | 65-78 | 25 |
| Tay[[122](#_ENREF_122)] | 2012 | Australia | 1 | 65 | 100 |
| Timbrell[[123](#_ENREF_123)] | 2012 | UK | 1 | 70 | 100 |
| Al-Abri[[124](#_ENREF_124)] | 2013 | US | 1 | 15 | 0 |
| Al-Abri[[125](#_ENREF_125)] | 2013 | US | 1 | 31 | 100 |
| Avci[[126](#_ENREF_126)] | 2013 | Turkey | 5 | 17-29 | 40 |
| Bein[[127](#_ENREF_127)] | 2013 | Besancon | 1 | 58 | 100 |
| Duger[[128](#_ENREF_128)] | 2013 | Turkey | 1 | 35 | 0 |
| Duong[[129](#_ENREF_129)] | 2013 | Australia | 14 | 45-90 | 35.7 |
| Hojer[[130](#_ENREF_130)] | 2013 | Sweden | 1 | 66 | 100 |
| Kalantar-Zadeh[[131](#_ENREF_131)] | 2013 | US | 1 | 54 | 0 |
| Kopec[[132](#_ENREF_132)] | 2013 | US | 1 | 39 | 100 |
| Kwok[[133](#_ENREF_133)] | 2013 | Hong Kong | 1 | 86 | 0 |
| Pasquel[[134](#_ENREF_134)] | 2013 | US | 2 | 56-70 | 100 |
| Plumb[[135](#_ENREF_135)] | 2013 | UK | 1 | 66 | 0 |
| Seo[[136](#_ENREF_136)] | 2013 | Korea | 1 | 82 | 0 |
| Wang[[137](#_ENREF_137)] | 2013 | Taiwan | 1 | 61 | 100 |
| Westerbergh[[138](#_ENREF_138)] | 2013 | Sweden | 1 | 66 | 100 |
| Zoppellari[[139](#_ENREF_139)] | 2013 | Italy | 1 | 66 | 0 |
| Altun[[140](#_ENREF_140)] | 2014 | Turkey | 1 | 76 | 100 |
| Doepker[[141](#_ENREF_141)] | 2014 | US | 1 | 59 | 100 |
| Fremin[[142](#_ENREF_142)] | 2014 | US | 1 | 49 | 100 |
| Hussain[[143](#_ENREF_143)] | 2014 | Australia | 2 | 67-68 | 50 |
| Ncomanzi[[144](#_ENREF_144)] | 2014 | Australia | 1 | 66 | 0 |
| Pallavi[[145](#_ENREF_145)] | 2014 | US | 1 | 60 | 100 |

^*^NA: Not available

**Figure S1.** Flow diagram of study selection process.

- Records identified through Medline/PubMed (n=108) and EMBASE (n=325)
- Records identified through manual search (n=4)

Duplicate records removed (n=94)

Records Screened

(n= 343)

***Records removed based on exclusion criteria*** (n=90)

- Not-metformin related lactic acidosis (e.g., phenformin and buformin)
- Not relevant to research questions
- Editorial, non-systemic reviews, and medial guidelines

Records eligible for compete review

(n=253)

***Records removed based on secondary exclusion criteria*** (n=108)

- Original articles, reviews, letters, comments, case-series or correspondences not providing patient-level data
- Conference abstracts not providing patient-level data
- Metformin was not the only offending factor resulting in lactic acidosis
- Survival status of an individual patient could not be ascertained
- Cases were not received renal replacement therapy

**Studies included in qualitative synthesis (n= 145)**

**Language**

English (n=115)

Spanish (n=10)

French (n=7)

Deutsch (n=5)

Danish (n=3)

Swedish (n=2)

Turkish (n=2)

Japanese (n=1)

**Figure S2.** The diagram of the definitions and coding rules of key variables.

**
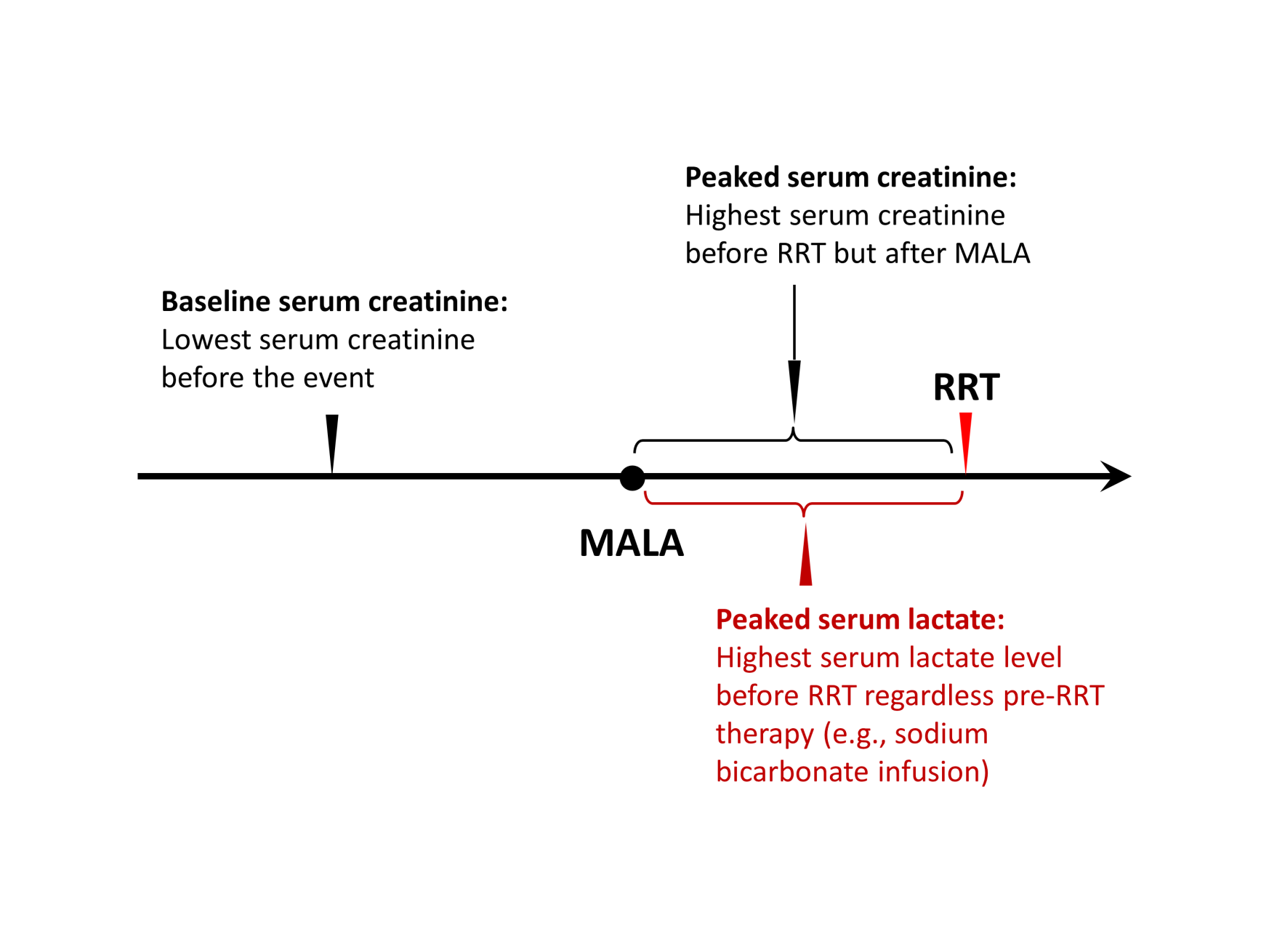
**

**Figure S3.** The distribution of number of published case reports by countries.


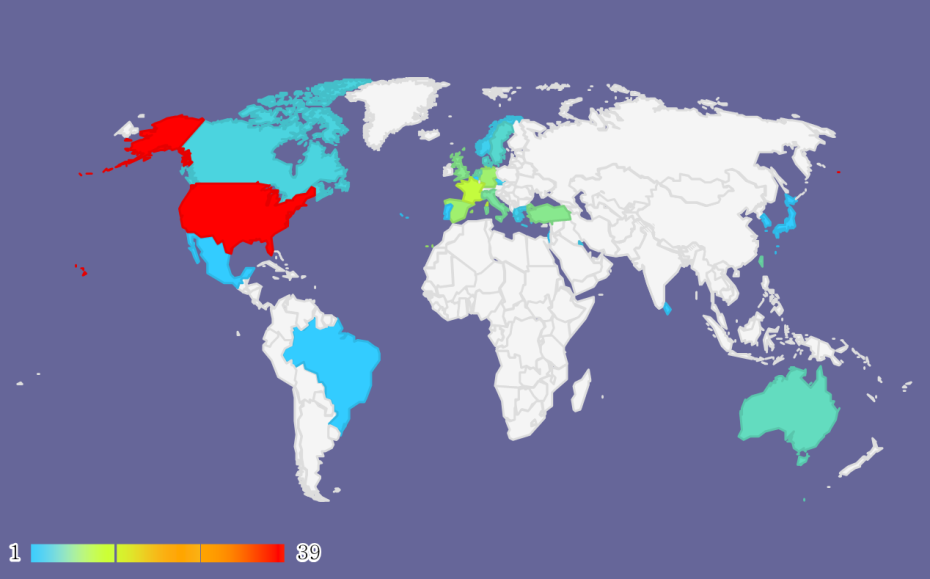


**Figure S4.** The detailed summary of disease course of a 50-year-old diabetic woman with severe metformin-associated lactic acidosis (MALA) after suicidal metformin ingestion of more than 40 grams treated at China Medical University Hospital in Taiwan. Disturbance of consciousness developed a few hours later followed by profound shock status. The initial investigation at our hospital revealed severe metabolic acidosis and a total of 622.5 mmol sodium bicarbonate was administered. However, due to refractory shock status and concomitant kidney failure, the patient was referred to our hospital. An 8-hour SLED-f was immediately conducted for her hypothermia(34℃), shock (systolic blood pressure 60 mmHg), hypernatremia (Na: 168 mmol/L), anuric kidney injury (serum creatinine 2.05 mg/dL), severe metabolic acidosis (pH: 6.84 and HCO3^-^: 4.4 mmol/L), and an extremely high serum lactate level to 55.5 mmol/L. The renal replacement therapy (RRT) modality was further switched to HVHF (70 ml/kg/hour) for refractory shock (maximal inotropic equivalent: 72 μg/kg/min) and lactic acidosis. Both SLED-f and HVHF were sequentially performed again in the first 48 hours of the disease course to maintain adequate pH. Although the total amount of sodium bicarbonate supplementation reached astronomically high levels (1660 mmol via intravenous infusion; 4400 mmol via replacement fluid), the serum sodium concentration could be maintained at normal range within 24 hours of RRT initiation. On the 4^th^ day of admission, the modality of RRT was downgraded to CVVH as we obtained a better control of acidosis. After the patient achieved hemodynarmic stability on the 6^th^ day of admission, the RRT was switched to intermittent hemodialysis (IHD) for an additional 14 days until the patient obtained independence from RRT. Her peak serum lactate level of 55.5mmol/l is more than any survived case we have reviewed in this systematic review. **Abbreviations:** Bicarb, each yellow rectangle of “Bicarb” stands for 250 ml of 7% sodium bicarbonate, which is comprised of 207.5mmol of Na^+^ and HCO3^-^; BT, body temperature; CVVH, continuous venovenous hemofiltration (replacement flow = 35 ml/kg/hour); HR, heart rate; HVHF, high volume hemofiltration (replacement flow＝70 ml/kg/hour); IE, inotropic equivalents; SBP, systolic blood pressure; SLED-f, sustained low-efficiency hemodiafiltration


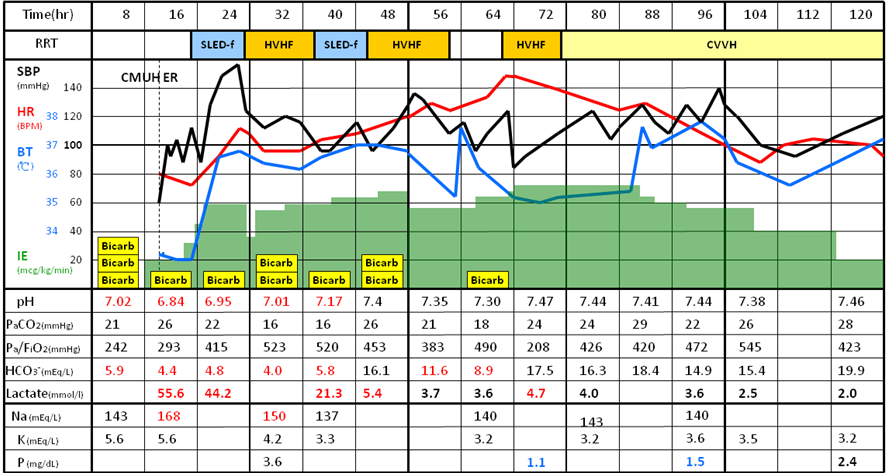


**References:**

1. Hayat JC: **The treatment of lactic acidosis in the diabetic patient by peritoneal dialysis using sodium acetate. A report of two cases**. *Diabetologia* 1974, **10**(5):485-487.

2. Bismuth C, Gaultier M, Conso F: **Lactate acidosis induced by excessive metformine ingestion**. *European Journal of Toxicology and Environmental Hygiene* 1976, **9**(1):55-57.

3. Assan R, Heuclin C, Ganeval D: **Metformin induced lactic acidosis in the presence of acute renal failure**. *Diabetologia* 1977, **13**(3):211-217.

4. Larcan A, Lambert H, Laprevote-Heully MC: **The treatment of lactic acidoses with hemodialysis upon polyacrylonitrile membrane**. *Annales Medicales de Nancy et de l'Est* 1981, **20**(AUG.-SEPT.):989-996.

5. Chalopin JM, Tanter Y, Besancenot JF: **Treatment of metformin-associated lactic acidosis with closed recirculation bicarbonate-buffered hemodialysis**. *Archives of Internal Medicine* 1984, **144**(1):203-205.

6. Perrot D, Claris O, Guillaume C, Bouffard Y, Delafosse B, Motin J: **Metformin and lactic acidosis: value of initial plasma assay of metformin and hemodialysis**. *Annales de médecine interne* 1986, **137**(2):169-170.

7. Huguet C, Lanotte R, Ged E: **Treatment of acute poisoning with a biguanide derivative (metformin)**. *Revue de Medecine de Tours* 1986, **20**(8):521-524.

8. Lalau JD, Westeel PF, Debussche X, Dkissi H, Tolani M, Coevoet B, Temperville B, Fournier A, Quichaud J: **Bicarbonate haemodialysis: An adequate treatment for lactic acidosis in diabetics treated by metformin**. *Intensive Care Medicine* 1987, **13**(6):383-387.

9. Lacroix C, Hermelin A, Gerson M, Nouveau J, Guiberteau R: **Lactic acidosis ascribable to metformin**. *Presse Medicale* 1988, **17**(22):1158-1158.

10. Khan IH, Catto GR, MacLeod AM: **Severe lactic acidosis in patient receiving continuous ambulatory peritoneal dialysis**. *BMJ (Clinical research ed)* 1993, **307**(6911):1056-1057.

11. Kovacs KA, Morton AR: **Metformin-associated lactic acidosis**. *Canadian Journal of Clinical Pharmacology* 1996, **3**(2):90-92.

12. Pearlman BL, Fenves AZ, Emmett M: **Metformin-associated lactic acidosis**. *American Journal of Medicine* 1996, **101**(1):109-110.

13. Heaney D, Majid A, Junor B: **Bicarbonate haemodialysis as a treatment of metformin overdose**. *Nephrology, dialysis, transplantation : official publication of the European Dialysis and Transplant Association - European Renal Association* 1997, **12**(5):1046-1047.

14. Jurovich MR, Wooldridge JD, Force RW: **Metformin-associated nonketotic metabolic acidosis**. *The Annals of pharmacotherapy* 1997, **31**(1):53-55.

15. Mercker SK, Maier C, Neumann G, Wulf H: **Lactic acidosis as a serious perioperative complication of antidiabetic biguanide medication with metformin**. *Anesthesiology* 1997, **87**(4):1003-1005.

16. Schmidt R, Horn E, Richards J, Stamatakis M: **Survival after metformin-associated lactic acidosis in peritoneal dialysis-dependent renal failure**. *American Journal of Medicine* 1997, **102**(5):486-488.

17. Darwich MS: **Metabolic acidosis**. *Postgraduate Medical Journal* 1998, **74**(873):427-428.

18. Gainza FJ, Gimeno I, Muniz R: **Metformin-associated lactic acidosis: Treatment with hemodialysis**. *Nefrologia* 1998, **18**(5):427-430.

19. Teale KFH, Devine A, Stewart H, Harper NJH: **The management of metformin overdose**. *Anaesthesia* 1998, **53**(7):698-701.

20. Stefansson B, Larsson B, Attman PO: **Hemodialysis cured severe lactic acidosis caused by metformin treatment**. *Läkartidningen* 1999, **96**(50):5622-5624.

21. Houwerzijl EJ, Snoek WJ, Van Haastert M, Holman ND: **Severe lactic acidosis after use of metformin in a patient with contraindications to metformin**. *Nederlands Tijdschrift voor Geneeskunde* 2000, **144**(40):1923-1926.

22. Reeker W, Schneider G, Felgenhauer N, Tempel G, Kochs E: **Metformin-induced lactacidosis**. *Deutsche Medizinische Wochenschrift* 2000, **125**(9):249-251.

23. Doorenbos CJ, Bosma RJ, Lamberts PJ: **Use of urea containing dialysate to avoid disequilibrium syndrome, enabling intensive dialysis treatment of a diabetic patient with renal failure and severe metformin induced lactic acidosis**. *Nephrology, dialysis, transplantation : official publication of the European Dialysis and Transplant Association - European Renal Association* 2001, **16**(6):1303-1304.

24. Ellis AK, Iliescu EA: **Metformin-associated lactic acidosis in a low risk patient**. *Canadian Journal of Clinical Pharmacology* 2001, **8**(2):104-106.

25. Kruse JA: **Metformin-associated lactic acidosis**. *Journal of Emergency Medicine* 2001, **20**(3):267-272.

26. Barrueto F, Meggs WJ, Barchman MJ: **Clearance of metformin by hemofiltration in overdose**. *Journal of Toxicology - Clinical Toxicology* 2002, **40**(2):177-180.

27. Berner B, Hummel KM, Strutz F, Ritzel U, Ramadori G, Hagenlocher S, Kleine P, Muller GA: **Metformin-induced lactic acidosis with acute renal failure in type 2 diabetes mellitus**. *Medizinische Klinik* 2002, **97**(2):99-103.

28. Chang CT, Chen YC, Fang JT, Huang CC: **Metformin-associated lactic acidosis: Case reports and literature review**. *Journal of Nephrology* 2002, **15**(4):398-402.

29. Chu CK, Chang YT, Lee BJ, Hu SY, Hu WH, Yang DY: **Metformin-associated lactic acidosis and acute renal failure in a type 2 diabetic patient**. *Journal of the Chinese Medical Association* 2003, **66**(8):505-508.

30. Gjedde S, Christiansen A, Pedersen SB, Rungby J: **Survival following a metformin overdose of 63 g: A case report**. *Pharmacology and Toxicology* 2003, **93**(2):98-99.

31. Heras M, Mon C, Sanchez R, Fernandez-Reyes MJ: **Kidney hypoperfusion and overdose of metformin as cause of severe lactic acidosis**. *Nefrología : publicación oficial de la Sociedad Española Nefrologia* 2003, **23**(5):465-466.

32. Nisse P, Mathieu-Nolf M, Deveaux M, Forceville X, Combes A: **A Fatal Case of Metformin Poisoning [3]**. *Journal of Toxicology - Clinical Toxicology* 2003, **41**(7):1035-1036.

33. Pertek JP, Vidal S, Mariot J, Galy-Floc'h M, Azoulay E: **Metformin-associated lactic acidosis precipitated by acute renal failure**. *Annales Francaises d'Anesthesie et de Reanimation* 2003, **22**(5):457-460.

34. Price G: **Metformin lactic acidosis, acute renal failure and rofecoxib**. *British Journal of Anaesthesia* 2003, **91**(6):909-910.

35. Schure PJ, de Gooijer A, van Zanten AR: **Unexpected survival from severe metformin-associated lactic acidosis**. *The Netherlands journal of medicine* 2003, **61**(10):331-333.

36. Iwai H, Ohno Y, Itoh H, Endo T, Komaki K, Ishii S, Morioka Y, Imonawa H, Kiyokawa T, Harada T *et al*: **Type 2 diabetes mellitus with lactic acidosis and acute renal failure induced by metformin overdose in suicide**. *Journal of the Japan Diabetes Society* 2004, **47**(6):439-445.

37. Mallick S: **Metformin induced acute pancreatitis precipitated by renal failure**. *Postgraduate Medical Journal* 2004, **80**(942):239-240.

38. Moerer O, Barwing J, Neumann P: **Metformin-associated lactic acidosis**. *Anaesthesist* 2004, **53**(2):153-156.

39. Pena JM, Pernaute R, Vicente C: **Acute kidney failure and severe lactic acidosis caused by metformin successfully treated with hemodialysis**. *Nefrología : publicación oficial de la Sociedad Española Nefrologia* 2004, **24**(1):89-90.

40. Panzer U, Kluge S, Kreymann G, Wolf G: **Combination of intermittent haemodialysis and high-volume continuous haemofiltration for the treatment of severe metformin-induced lactic acidosis [5]**. *Nephrology Dialysis Transplantation* 2004, **19**(8):2157-2158.

41. Von Mach MA, Sauer O, Sacha Weilemann L: **Experiences of a poison center with metformin-associated lactic acidosis**. *Experimental and Clinical Endocrinology and Diabetes* 2004, **112**(4):187-190.

42. Harvey B, Hickman C, Hinson G, Ralph T, Mayer A: **Severe lactic acidosis complicating metformin overdose successfully treated with high-volume venovenous hemofiltration and aggressive alkalinization**. *Pediatric Critical Care Medicine* 2005, **6**(5):598-601.

43. Lacher M, Hermanns-Clausen M, Haeffner K, Brandis M, Pohl M: **Severe metformin intoxication with lactic acidosis in an adolescent**. *European Journal of Pediatrics* 2005, **164**(6):362-365.

44. Runge S, Warnke C, Abel P, Friesecke S, Felix S: **Lethal metformin-associated lactic acidosis in a patient with acute decompensation of chronic renal failure**. *Intensivmedizin und Notfallmedizin* 2005, **42**(2):172-176.

45. Clare S, Paul P, Hulley C, Jones S: **Metformin associated lactic acidosis not as rare as we think?** *Acute Medicine* 2006, **5**(4):99-101.

46. Friesecke S, Abel P, Kraft M, Gerner A, Runge S: **Combined renal replacement therapy for severe metformin-induced lactic acidosis [18]**. *Nephrology Dialysis Transplantation* 2006, **21**(7):2038-2039.

47. Gudmundsdottir H, Aksnes H, Heldal K, Krogh A, Froyshov S, Rudberg N, Os I: **Metformin and antihypertensive therapy with drugs blocking the renin angiotensin system, a cause of concern?** *Clinical Nephrology* 2006, **66**(5):380-385.

48. Guo PY, Storsley LJ, Finkle SN: **Severe lactic acidosis treated with prolonged hemodialysis: recovery after massive overdoses of metformin**. *Semin Dial* 2006, **19**(1):80-83.

49. Aquarius M, Van Kuijk WHM: **Fatal autointoxication with metformin [3]**. *Nederlands Tijdschrift voor Geneeskunde* 2007, **151**(32):1809.

50. El-Hennawy AS, Jacob S, Mahmood AK: **Metformin-associated lactic acidosis precipitated by diarrhea**. *American Journal of Therapeutics* 2007, **14**(4):403-405.

51. Galea M, Jelacin N, Bramham K, White I: **Severe lactic acidosis and rhabdomyolysis following metformin and ramipril overdose**. *British Journal of Anaesthesia* 2007, **98**(2):213-215.

52. Gonzalez Losada T, Alcazar V, De Icaya PM, Del Olmo D, Del Val TL, Perez M, Herranz S: **A case of lactic acidosis related to acute renal failure and metformin intake treated with bicarbonate haemodialysis**. *Endocrinologia y Nutricion* 2007, **54**(9):496-499.

53. De La Maza Pereg L, Hierro VMG, Banos PA, Barrio JP, Lopez LC, Perez ER: **Metformin-induced lactic acidosis**. *Endocrinologia y Nutricion* 2007, **54**(6):325-327.

54. Lopez JC, Esteve F, Jubert E, Villanueva H, Perez XL: **Metformin-associated lactic acidosis. Combined treatment with haemodialysis and continuous hemofiltration**. *Endocrinologia y Nutricion* 2007, **54**(9):500-503.

55. Radej J, Matejovic M, Krouzecky A, Sykora R, Chvojka J, Novak I: **How severe acidosis can a human survive? Successful hemofiltration use**. *Dialysis and Transplantation* 2007, **36**(11):608-610.

56. Shenoy C: **Metformin-associated lactic acidosis precipitated by acute renal failure**. *The American journal of the medical sciences* 2006, **331**(1):55-57.

57. Silvestre J, Carvalho S, Mendes V, Coelho L, Tapadinhas C, Ferreira P, Povoa P, Ceia F: **Metformin-induced lactic acidosis: A case series**. *Journal of Medical Case Reports* 2007, **1**.

58. Almirall J, Briculle M, Gonzalez-Clemente JM: **Metformin-associated lactic acidosis in type 2 diabetes mellitus: incidence and presentation in common clinical practice**. *Nephrology, dialysis, transplantation : official publication of the European Dialysis and Transplant Association - European Renal Association* 2008, **23**(7):2436-2438.

59. Audia P, Feinfeld DA, Dubrow A, Winchester JF: **Metformin-induced lactic acidosis and acute pancreatitis precipitated by diuretic, celecoxib, and candesartan-associated acute kidney dysfunction**. *Clinical Toxicology* 2008, **46**(2):164-166.

60. Balik M, Waldauf P, Glocknerova K, Kusova D: **Lactate-buffered dialysis in cardiogenic shock associated with severe combined lactic acidosis**. *NDT Plus* 2008, **1**(2):103-105.

61. Bruijstens LA, van Luin M, Buscher-Jungerhans PM, Bosch FH: **Reality of severe metformin-induced lactic acidosis in the absence of chronic renal impairment**. *The Netherlands journal of medicine* 2008, **66**(5):185-190.

62. Di Grande A, Vancheri F, Giustolisi V, Giuffrida C, Narbone G, Licata M, Le Moli C, Riccobene S, Burgio A, Bartolotta S *et al*: **Metformin-induced lactic acidosis in a type 2 diabetic patient with acute renal failure**. *Clinica Terapeutica* 2008, **159**(2):87-89.

63. Khan FY, Ibrahim AS, Errayes M: **Life threatening lactic acidosis secondary to metformin, in a low risk patient**. *Journal of Clinical and Diagnostic Research* 2008, **2**(2):754-756.

64. Redha F, Al-Shemmeri M, Ibrahim H: **Metformin induced lactic acidosis in a patient with anorexia nervosa: A case report and literature review**. *Kuwait Medical Journal* 2008, **40**(3):230-232.

65. Teutonico A, Libutti P, Lomonte C, Antonelli M, Casucci F, Basile C: **Treatment of metformin-associated lactic acidosis with sustained low-efficiency daily dialysis**. *NDT Plus* 2008, **1**(5):380-381.

66. Abramo A, Corini M, Malacarne P, Donadio F, Donadio C: **Early and prolonged continuous hemodiafi ltration for the treatment of severe metformin-associated lactic acidosis**. *Dialysis and Transplantation* 2009, **38**(4):139-144.

67. Barbani F, Di Filippo A, Linden M, Pasquini A, Cammelli R, Cianchi G, Peris A: **Metformin-associated lactic acidosis: Three patients surviving after continuous venovenous haemofiltration**. *Critical Care* 2009, **13**:S198.

68. Brouwers MC, Schaper N, Keeris L: **Does glucose infusion exacerbate metformin-associated lactate acidosis? A case report**. *Diabetes Res Clin Pract* 2009, **85**(1):e1-3.

69. Chang LC, Hung SC, Yang CS: **The case | A suicidal woman with delayed high anion gap metabolic acidosis**. *Kidney International* 2009, **75**(7):757-758.

70. Dora JM, De Souza LH, De Azevedo MJ, Gross JL: **Lactic acidosis associated with metformin in a patient with type 2 diabetes with no contraindication**. *Endocrinologist* 2009, **19**(6):250.

71. Fitzgerald E, Mathieu S, Ball A: **Lesson of the week: Metformin associated lactic acidosis**. *BMJ (Online)* 2009, **339**(7732):1254-1256.

72. Hagset IB, Krogh AV, Froyshov S: **Life-threatening lactic acidosis in a patient using therapeutic doses of metformin and ACE-inhibitor**. *Clinical Toxicology* 2009, **47**(5):497-498.

73. Kreshak AA, Clark RF: **Vision loss in a patient with metformin-associated lactic acidosis**. *Clinical Toxicology* 2009, **47**(7):726.

74. Leung S, Kent R, Sivaraman S, Ali F: **Rapid restoration of hemodynamic stability in patients with metformin-assoicated lactic acidosis after renal replacement therapy**. *Critical Care Medicine* 2009, **37**(12):A521.

75. Mizzi A, Landoni G, Corno L, Fichera M, Nuzzi M, Zangrillo A: **How to explain a PaO2 of 140 mmHg in a venous line?** *Acta Biomedica de l'Ateneo Parmense* 2009, **80**(3):262-264.

76. Pan LT, MacLaren G: **Continuous venovenous haemodiafiltration for metformin-induced lactic acidosis**. *Anaesth Intensive Care* 2009, **37**(5):830-832.

77. Turkcuer I, Erdur B, Sari I, Yuksel A, Tura P, Yuksel S: **Severe metformin intoxication treated with prolonged haemodialyses and plasma exchange**. *European Journal of Emergency Medicine* 2009, **16**(1):11-13.

78. Wen YK: **Impact of acute kidney injury on metformin-associated lactic acidosis**. *International Urology and Nephrology* 2009, **41**(4):967-972.

79. Yang PW, Lin KH, Lo SH, Wang LM, Lin HD: **Successful treatment of severe lactic acidosis caused by a suicide attempt with a metformin overdose**. *Kaohsiung Journal of Medical Sciences* 2009, **25**(2):93-97.

80. Zur Nieden T, Conrad T: **Female patient with type 2 diabetes in coma with metabolic acidosis**. *Internist* 2009, **50**(11):1275-1280.

81. Arroyo AM, Walroth TA, Mowry JB, Kao LW: **The MALAdy of metformin poisoning: Is CVVH the cure?** *American Journal of Therapeutics* 2010, **17**(1):96-100.

82. Carrillo Esper R, Sosa Garcia JO: **Lactic acidosis due to metformin**. *Medicina Interna de Mexico* 2010, **26**(3):276-280.

83. Dell'Aglio DM, Perino LJ, Todino JD, Algren DA, Morgan BW: **Metformin Overdose With a Resultant Serum pH of 6.59: Survival Without Sequalae**. *Journal of Emergency Medicine* 2010, **39**(1):e77-e80.

84. Gamst J, Hansen LK, Rasmussen BS: **[Metformin treatment causes persisting lactic acidosis after cardiac arrest]**. *Ugeskr Laeger* 2010, **172**(49):3418-3419.

85. Giuliani E, Albertini G, Vaccari C, Barbieri A: **pH 6.68-surviving severe metformin intoxication**. *QJM* 2010, **103**(11):887-890.

86. Huberlant V, Laterre PF, Hantson P: **Nearly fatal metabolic acidosis: Septic or toxic?** *European Journal of Emergency Medicine* 2010, **17**(4):243-244.

87. Kavalci C, Guldiken S, Taskiran B: **Fatal lactic acidosis due to metformine**. *Internet Journal of Internal Medicine* 2010, **8**(1).

88. Livshits Z, Nelson LS, Hernandez SH, Smith SW, Howland MA, RS H: **Severe Metformin Toxicity: Role of Methylene Blue and CVVHD as Therapeutic Adjuncts.** In: *2010 North American Congress of Clinical Toxicology.* Denver, CO; 2010: Abstract 36.

89. Olivera-Gonzalez S, de Escalante-Yanguela B, Velilla-Soriano C, Amores-Arriaga B, Martin-Fortea P, Navarro-Aguilar ME: **Metformin-associated hepatotoxicity**. *Medicina Intensiva* 2010, **34**(7):483-487.

90. Orsi D, Dudaie R, Dicpinigaitis P: **Acute pancreatitis associated with metformin toxicity**. *Critical Care Medicine* 2010, **38**:A271.

91. Thajeb P, Thajeb T, Dai DF: **Metformin induces fulminant lactic acidosis and fatal hepatorenal syndrome in a patient with diabetes mellitus due to mitochondrial disease**. *European Journal of Neurology* 2010, **17**:605.

92. Walsh SJ, Abesamis MG, Cannon RD: **Severe metformin-associated lactic acidosis from acute ingestion without renal failure**. *Clinical Toxicology* 2010, **48**(6):614.

93. Akoglu H, Akan B, Piskinpasa S, Karaca O, Dede F, Erdem D, Albayrak MD, Odabas AR: **Metformin-associated lactic acidosis treated with prolonged hemodialysis**. *American Journal of Emergency Medicine* 2011, **29**(5):575.e573-575.e575.

94. Aperis G, Paliouras C, Zervos A, Arvanitis A, Alivanis P: **Lactic acidosis after concomitant treatment with metformin and tenofovir in a patient with HIV infection**. *Journal of Renal Care* 2011, **37**(1):25-29.

95. Chow LH, Shah N, Pham T: **Acute vision loss and lactic acidosis**. *Journal of General Internal Medicine* 2011, **26**:S546.

96. Cosenza L, Al-Dahir S, Engel LS, Nielsen N: **A potentially fatal case of mistaken identity: Metformin vs oxycodone-acetaminophen**. *Journal of Investigative Medicine* 2011, **59**(2):385.

97. Devetzis V, Passadakis P, Panagoutsos S, Theodoridis M, Thodis E, Georgoulidou A, Vargemezis V: **Metformin-related lactic acidosis in patients with acute kidney injury**. *International Urology and Nephrology* 2011, **43**(4):1243-1248.

98. Martin Gómez MA, Sánchez Martos MD, García Marcos SA, Serrano Carrillo de Albornoz JL: **Acidosis láctica grave por metformina: utilidad de la medición de niveles y terapia con hemodiálisis de alto flujo**. *Nefrología (Madrid)* 2011, **31**:610-611.

99. Hellervik SM, Wheeler AP, Burgner A: **Metformin associated lactic acidosis**. *Critical Care Medicine* 2011, **39**:256.

100. Jagia M, Taqi S, Hanafi M: **Metformin poisoning: A complex presentation**. *Indian Journal of Anaesthesia* 2011, **55**(2):190-192.

101. Keller G, Cour M, Hernu R, Illinger J, Robert D, Argaud L: **Management of metformin-associated lactic acidosis by continuous renal replacement therapy**. *PLoS ONE* 2011, **6**(8).

102. Miller SN, Greenberg MI: **Failure of lipid emulsion therapy to treat a metformin overdose**. *Clinical Toxicology* 2011, **49**(6):538-539.

103. Miller DK, Brinson AJ, Catalano G, Catalano MC: **Lactic acidosis, hypotension, and sensorineural hearing loss following intentional metformin overdose**. *Current Drug Safety* 2011, **6**(5):346-349.

104. Parekh N, Soi S: **Osmolar gap metabolic acidosis associated with lactic acidosis in patient taking metformin: A case report**. *American Journal of Kidney Diseases* 2011, **57**(4):A76.

105. Parke C, Lien YH: **Quiz page June 2011. Profound metabolic acidosis and abdominal pain in a diabetic patient on long-term hemodialysis**. *American journal of kidney diseases : the official journal of the National Kidney Foundation* 2011, **57**(6):A25-27.

106. Perrone J, Phillips C, Gaieski D: **Occult metformin toxicity in three patients with profound lactic acidosis**. *Journal of Emergency Medicine* 2011, **40**(3):271-275.

107. Pikwer A, Vernersson E, Frid A, Sterner G: **Extreme lactic acidosis type B associated with metformin treatment**. *NDT Plus* 2011, **4**(6):399-401.

108. Rawat N, Hincapie J, Ramirez N: **Metformin toxicity presenting with severe met-abolicacidosis, blindnessand renalfailure mimicking methanol poisoning**. *Critical Care Medicine* 2011, **39**:245.

109. Rifkin SI, McFarren C, Juvvadi R, Weinstein SS: **Prolonged hemodialysis for severe metformin intoxication**. *Renal Failure* 2011, **33**(4):459-461.

110. Sencan A, Adanir T, Atay A, Atasoy AA, Aksun M, Terzi G, Karahan N: **High anion gap metabolic acidosis after suicide: Metformin intoxication**. *Anestezi Dergisi* 2011, **19**(1):56-59.

111. Soyoral YU, Begenik H, Emre H, Aytemiz E, Ozturk M, Erkoc R: **Dialysis therapy for lactic acidosis caused by metformin intoxication: Presentation of two cases**. *Human and Experimental Toxicology* 2011, **30**(12):1995-1997.

112. Surani S, Morales M, Rodriguez M, Varon J: **The resilience of the human body**. *American Journal of Emergency Medicine* 2011, **29**(7):835-837.

113. Yeung CW, Chung HY, Fong BM, Tsai NW, Chan WM, Siu TS, Tam S, Tsui SH: **Metformin-associated lactic acidosis in Chinese patients with type II diabetes**. *Pharmacology* 2011, **88**(5-6):260-265.

114. Baro-Serra A, Guasch-Aragay B, Martin-Alemany N, Sirvent JM, Valles-Prats M: **The importance of early haemodiafiltration in the treatment of lactic acidosis associated with the administration of metformin**. *Nefrologia* 2012, **32**(5):664-669.

115. Dichtwald S, Weinbroum AA, Sorkine P, Ekstein MP, Dahan E: **Metformin-associated lactic acidosis following acute kidney injury. Efficacious treatment with continuous renal replacement therapy**. *Diabetic Medicine* 2012, **29**(2):245-250.

116. Jorgensen HK, Nielsen JS, Gilsaa T: *Ugeskrift for Laeger* 2012, **174**(23):1602-1603.

117. Montini F, Rondeau E, Peltier J, Mesnard L, Jouzel C, Ridel C: **[Metformin associates lactic acidosis]**. *Presse Med* 2012, **41**(10):907-916.

118. Mujtaba M, Geara AS, Madhrira M, Agarwala R, Anderson H, Cheng JT, Mohan S: **Toxicokinetics of metformin-associated lactic acidosis with continuous renal replacement therapy**. *European Journal of Drug Metabolism and Pharmacokinetics* 2012, **37**(4):249-253.

119. Mustafa E, Lai L, Lien YH: **Rapid recovery from acute kidney injury in a patient with metformin-associated lactic acidosis and hypothermia**. *Am J Med* 2012, **125**(2):e1-2.

120. Rathnapala A, Matthias T, Jayasinghe S: **Severe lactic acidosis and acute renal failure following ingestion of metformin and kerosene oil: A case report**. *Journal of Medical Case Reports* 2012, **6**.

121. Schousboe K, El Fassi D, Secher EL, Elming H, Rasmussen K, Hornum M: *Ugeskrift for Laeger* 2012, **174**(23):1604-1606.

122. Tay S, Lee IL: **Survival after cardiopulmonary arrest with extreme hyperkalaemia and hypothermia in a patient with metformin-associated lactic acidosis**. *BMJ Case Reports* 2012.

123. Timbrell S, Wilbourn G, Harper J, Liddle A: **Lactic acidosis secondary to metformin overdose: A case report**. *Journal of Medical Case Reports* 2012, **6**.

124. Al-Abri SA, Hayashi S, Thoren KL, Olson KR: **Metformin overdose-induced hypoglycemia in the absence of other antidiabetic drugs**. *Clinical Toxicology* 2013, **51**(5):444-447.

125. Al-Abri SA, Thoren KL, Benowitz NL: **Metformin overdose-induced recurrent hypoglycemia**. *Clinical Toxicology* 2013, **51**(7):671-672.

126. Avci D, Cetinkaya A, Karahan S, Oguzhan N, Karagoz H, Basak M, Erden A: **Suicide commitment with metformin: Our experience with five cases**. *Renal Failure* 2013, **35**(6):863-865.

127. Bein C, Duroy E, Valette S, Gaiffe A, Davani S: **Metformin self poisoning: A case report**. *Fundamental and Clinical Pharmacology* 2013, **27**:76.

128. Duger C, Isbir AC, Ozdemir Kol I, Kaygusuz K, Gursoy S, Kilic I, Mimaroglu C: **Successful treatment of intoxication case with rosiglitasone and metformin**. *Anestezi Dergisi* 2013, **21**(3):190-193.

129. Duong JK, Furlong TJ, Roberts DM, Graham GG, Greenfield JR, Williams KM, Day RO: **The role of metformin in metformin-associated lactic acidosis (MALA): Case series and formulation of a model of pathogenesis**. *Drug Safety* 2013, **36**(9):733-746.

130. Hojer J, Westerbergh J, Edfeldt-Ugarph M, Johansson A: *Lakartidningen* 2013, **110**(42):1865.

131. Kalantar-Zadeh K, Uppot RN, Lewandrowski KB: **Case 23-2013: A 54-year-old woman with abdominal pain, vomiting, and confusion**. *New England Journal of Medicine* 2013, **369**(4):374-382.

132. Kopec KT, Kowalski MJ: **Metformin-Associated Lactic Acidosis (MALA): Case Files of the Einstein Medical Center Medical Toxicology Fellowship**. *Journal of Medical Toxicology* 2013, **9**(1):61-66.

133. Kwok WC, Chan TC, Luk J, Chan F: **Metformin-associated lactic acidosis in an older adult after colonoscopy: An uncommon trigger for a rare complication**. *Journal of the American Geriatrics Society* 2013, **61**(12):2257-2258.

134. Pasquel FJ, Klein R, Adigweme A, Hinedi Z, Coralli R, Pimentel JL, Umpierrez GE: **Metformin-associated lactic acidosis**. *The American journal of the medical sciences* 2015, **349**(3):263-267.

135. Plumb B, Parker A, Wong P: **Feeling blue with metformin-associated lactic acidosis**. *BMJ Case Reports* 2013.

136. Seo JH, Lee da Y, Hong CW, Lee IH, Ahn KS, Kang GW: **Severe lactic acidosis and acute pancreatitis associated with cimetidine in a patient with type 2 diabetes mellitus taking metformin**. *Internal medicine (Tokyo, Japan)* 2013, **52**(19):2245-2248.

137. Wang TH, Tsai WJ, Li YT, Wei-Lan C, Deng JF: **Severe metformin poisoning survived by continuous venovenous hemofiltration and extracorporeal membrane oxygenation**. *Clinical Toxicology* 2013, **51**(4):366.

138. Westerbergh J, Hojer J: **Metformin-induced refractory vasodilatory shock successfully treated with methylene blue**. *Clinical Toxicology* 2013, **51**(4):364-365.

139. Zoppellari R, Bortolazzi S, Petrini S, Verri M, Dallocchio G, Felisatti G, Petrini L, Bianchi S, Bertocco C, Avato FM: **A case of cardiac arrest related to therapeutic use of metformin: Clinical and toxicological aspects**. *Clinical Toxicology* 2013, **51**(4):365-366.

140. Altun E, Kaya B, Paydas S, Sariakcali B, Karayaylali I: **Lactic acidosis induced by metformin in a chronic hemodialysis patient with diabetes mellitus type 2**. *Hemodialysis International* 2014, **18**(2):529-531.

141. Doepker B, Healy W, Cortez E, Adkins EJ: **High-dose insulin and intravenous lipid emulsion therapy for cardiogenic shock induced by intentional calcium-channel blocker and Beta-blocker overdose: a case series**. *The Journal of emergency medicine* 2014, **46**(4):486-490.

142. Fremin K, Owen J: **Metformin overdose and early renal replacement therapy**. *American Journal of Kidney Diseases* 2014, **63**(5):A47.

143. Hussain MI, Hall BM, Depczynski B, Connor SJ: **Acute renal failure and metformin-associated lactic acidosis following colonoscopy**. *Diabetes Research and Clinical Practice* 2014, **105**(1):e6-e8.

144. Ncomanzi D, Sicat RMR, Sundararajan K: **Metformin-associated lactic acidosis presenting as an ischemic gut in a patient who then survived a cardiac arrest: A case report**. *Journal of Medical Case Reports* 2014, **8**(1).

145. Pallavi R, Chaudhari A: **Prolonged hemodialysis: An antidote for metformin-associated lactic acidosis**. *American Journal of Therapeutics* 2014.
